# Effects over time of parenting interventions to reduce physical and emotional violence against children: a systematic review and meta-analysis

**DOI:** 10.1016/j.eclinm.2023.102003

**Published:** 2023-05-18

**Authors:** Sophia Backhaus, Patty Leijten, Janina Jochim, G.J. Melendez-Torres, Frances Gardner

**Affiliations:** a Centre for Evidence-based Intervention, Department of Social Policy and Intervention, University of Oxford, 32 Wellington Square, OX1 2ER, Oxford, UK; b Research Institute Child Development and Education, University of Amsterdam, Nieuwe Achtergracht 127, Postbus 15776, 1001 NG, Amsterdam, the Netherlands; c Medical School, University of Exeter, Heavitree Road, EX1 2LU, Exeter, UK

**Keywords:** Violence against children, Systematic review, Parenting, Intervention, Meta analysis

## Abstract

**Background:**

Violence against children affects over one billion children globally. International organisations promote parenting interventions as a main strategy to reduce violence against children. Parenting interventions have therefore been implemented rapidly across the globe. Yet, evidence for their longer-term effects remains unclear. We integrated global evidence to estimate effects over time of parenting interventions to reduce physical and emotional violence against children.

**Methods:**

In this systematic review and meta-analysis, we searched 26 databases and trial registries (14 non-English: Spanish, Chinese, Farsi, Russian, Thai) and conducted an extensive grey literature search up to August 01, 2022. We included randomised controlled trials (RCTs) of parenting interventions based on social learning theory for parents of children aged 2–10 years, without time or context restrictions. We critically appraised studies using Cochrane's Risk of Bias Tool. Data were synthesised using robust variance estimation meta-analyses. This study is registered with PROSPERO, CRD42019141844.

**Findings:**

We screened 44,411 records and included 346 RCTs. Sixty RCTs reported outcomes on physical or emotional violence. Trials were distributed across 22 countries (22% LMICs). Risk of bias was high for various domains. Outcome data ranged from 0 weeks to 2 years after the intervention, and was largely based on parent self-report. Parenting interventions reduced physical and emotional violent parenting behaviours immediately after the intervention (n = 42, k = 59; *d* = −0.46; 95% CI: −0.59, −0.33), at 1–6 months follow-up (n = 18, k = 31; *d* = −0.24; 95% CI: −0.37, −0.11) and at 7–24 months follow-up (n = 12, k = 19; *d* = −0.18; 95% CI: −0.34, −0.02), but effects were smaller over time.

**Interpretation:**

Our findings suggest that parenting interventions can reduce physical and emotional violence against children. Effects are maintained up to 24 months follow-up, but with diminished effect sizes. With global policy interest and imminent importance, research beyond 2 years and how effects can be better sustained over time is urgently needed.

**Funding:**

Student scholarship from the 10.13039/501100000269Economic Social Research Council, Clarendon, and Wolfson Isaiah Berlin Fund.

**Research in context**

**Evidence before this study**

Previous systematic reviews examining the effects of parenting interventions on child maltreatment found small significant effects. However, only two reviews conducted preliminary analyses on whether effects sustain over time and did not provide average effects at varying length of follow-up. Therefore, we systematically searched 26 databases and trial registries in English, Spanish, Chinese, Farsi, Russian and Thai with no language restrictions from inception to August 01, 2022, using search terms “intervention”, “parenting”, and “child behavior” or “violence”, and synonyms, for randomised controlled trials (RCTs) of parenting interventions based on social learning theory for parents of children aged 2–10 years.

**Added value of this study**

To our knowledge, this is the first systematic review that aimed to meta-analyse the effects of parenting intervention on physical and emotional violence against children at various time points after participation. We included all available evidence from randomised trials on physical and emotional parenting behaviours that are known to hurt children, including over fifty years of research from across the globe. From 346 eligible RCTs, 60 provided data on physical and emotional violence after participation in a parenting programme. We found that parenting interventions based on social learning theory are effective in reducing physical and emotional violence at immediate post-test, but effects are smaller at 1–6 months and 7–24 months follow-up. Risk of bias varied strongly between studies.

**Implications of all the available evidence**

Our findings suggest that effects of parenting programmes on physical and emotional violence decrease over time. With global policy interest and rapid scale-up of parenting interventions, it is crucial to understand how the magnitude of effects can be sustained over time. Due to a lack of studies, long-term effects of parenting intervention to reduce violence remain unclear.

## Introduction

Violence against children is a global public health and costly societal problem with devastating consequences to child development and health.^1^ Globally, an estimated one billion children experience violence.^2^ The COVID-19 pandemic has only exacerbated the situation for children by increasing risk factors for violence such as financial instability.^3^ Violence against children not only violates the United Nations Convention on the Rights of the Child^4^ but its prevention is also recognised as a key global goal as featured in the Sustainable Development Goals.^5^ In collaboration with other partners (e.g., UNICEF, CDC), the World Health Organization (WHO) is promoting the use of evidence-based strategies to end violence against children.^6^ One key strategy is the support of parents and caregivers, because they are the main perpetrators of physical and emotional violence against children.^7^ Parenting interventions are therefore increasingly implemented at scale, with growing policy interest such as the recent publication of WHO Guidelines on parenting interventions to prevent child maltreatment and enhance parent-child relationships.^8^

Parenting interventions are behavioural interventions directed at parents or other caregivers of the child that typically focus on parents learning new parenting skills and behaviours to improve the way they relate to their child.^8^ These interventions aim to strengthen the quality of parent–child relationship and enhance parenting knowledge and competence (see Panel 1). They are predominantly grounded in operant and social learning theory,^9^ with the premise that children learn behaviours by modelling their parents and through rewards and punishment, and that violent behaviours are often unwittingly reinforced in coercive interactions.^10^ In a coercive cycle of parent-child interaction, child non-compliance provokes anger and hostility in the parent which leads to a punitive response. This parental response provokes and negatively reinforces child disruptive behaviours, to which the parent responses with even more harshness. This interaction then spirals to violent interactions between parents and children. Social learning theory-based interventions are expected to break these violent interactions between parent and children by teaching parents effective alternative behaviour management skills such as the reinforcement of positive child behaviours and non-violent disciplining techniques.

Panel 1 **Parenting interventions in a nutshell.8**

Parenting interventions aim to improve parents and primary caregivers’ parenting quality by teaching parents new and non-violent behaviours and skills to interact with their children. They typically consist of a structured series of sessions, are manualised, and delivered in group or individual formats in the home, community, health setting, or online.

Besides parenting behaviours, interventions may also address parental knowledge about child development, attitudes towards violent parenting such as spanking, parenting beliefs, and parenting self-efficacy.

Components of parenting interventions that have shown effective in reducing violent parenting include, for example:

• ignoring negative child behaviours to elicit attention;
• using logical consequences (e.g., losing privileges);
• praising and rewarding appropriate child behaviours;
• improving parental self-management skills such as emotion-regulation.

These components are based on social learning theory that posits that children learn disruptive behaviours when parents unwittingly reward these behaviours and model aversive, often violent, behaviours.

Evidence suggests that parenting interventions reduce violent parenting behaviours. Most reviews found small effects,^11^, ^12^, ^13^, ^14^, ^15^, ^16^, ^17^ thus, one meta-analysis including only randomised controlled trials (RCTs) found no effect after controlling for publication bias.^18^ Existing meta-analyses vary widely in how they define child maltreatment, making it difficult to compare their findings and draw conclusions about intervention effects. While some meta-analyses include proxies of violence against children such as correlates of child abuse or general risk for violence,^12^, ^13^, ^14^ others include only validated child maltreatment instruments or official reports.^11^^,^^18^ In the present study, we include all physical and emotional parenting behaviours known to harm children's well-being and development, regardless of whether the primary studies labelled them as maltreatment or violence against children, or not. Theoretically, this is in line with various United Nations frameworks that increasingly included any form of physical and emotionally harmful parenting in their definition of violence against children.^1^^,^^19^ Empirically, it is in line with findings that measures of harsh parenting share on average 73% of their parenting behaviours with validated child maltreatment instruments.^20^

Most meta-analyses only studied immediate effects of parenting interventions on violence against children. Studying longer-term effects is not common practice, due to ethical and financial challenges, such as wait-list control groups or limited means to collect follow-up data. But because the main aim of parenting interventions is sustained change in parenting behaviours, the true effect of interest is the effects of parenting interventions over time. With parenting interventions increasingly going to scale, this knowledge becomes urgent. Prior reviews, conducted when the evidence base was smaller, and limited to searching English language databases, suggest sustained beneficial effects on child maltreatment.^13^^,^^18^

In the present systematic review and meta-analysis, we examined the effects of parenting interventions based on social learning theory to reduce violence against children over time by examining effects at different follow-up times. We focus on social learning theory-based programmes since these programmes are the most widely implemented and scaled-up parenting programmes.

## Methods

### Search strategy

We report this systematic review and meta-analysis according to the Preferred Reporting Items for Systematic reviews and Meta-Analyses guidelines and used Cochrane guidance for systematic reviews of interventions.^21^^,^^22^ We searched for trials in three ways. First, we included trials from our systematic review completed in 2014 that used the same inclusion and exclusion criteria.^23^ Second, we included eligible trials from our recent systematic review that covered studies from low- and middle-income countries and deployed a comprehensive search strategy with an exhaustive grey literature and multi language search in English, Thai, Spanish, Chinese, Farsi, and Russian (CRD42018088697; search updated in August 2022). Third, we systematically searched for eligible trials in 11 databases between January 01, 2014 and August 01, 2022 (3ie Database of Impact evaluations, ASSIA, Campbell Library, The Cochrane Library (Cochrane Database of Systematic Reviews, Cochrane Central Register of Controlled Trials), EMBASE, ERIC, MEDLINE, National Criminal Justice Reference Service, The International Bibliography of the Social Sciences, PsycINFO, PILOTS), and the following trial registries: ClinicalTrials.gov, Australian New Zealand Clinical Trials Registry, WHO International Clinical Trials Registry Platform, metaRegister of Controlled Trials (mRCT). We imposed no language restrictions. Search terms surrounded three conceptual categories: a. intervention, b. parenting, c. child behavioural and emotional problems or maltreatment/violence. In addition, we hand-searched the reference lists of 29 relevant systematic reviews identified in our search, and contacted authors by e-mail to request study results and unpublished manuscripts identified through trial registries. Two authors (SB, JJ) pilot tested the screening criteria on a random sample of 55 records. The first author (SB) screened 100% of the titles and abstracts and retrieved and screened all relevant full-text articles for eligibility. The third author (JJ) double-screened a random 20% of titles and abstracts, and a random 20% of full-texts. Finally, we checked the articles that met the inclusion criteria for duplicate reporting of the same data.

### Inclusion criteria

We included randomised-controlled trials with a no treatment, wait-list, minimal intervention, or care as usual control group. Parenting interventions were considered for inclusion when a minimum of 50% of sessions or content was directed at parents and the programme was guided by a strong social learning theoretical foundation (see Table S1 for more information). We did not place any restrictions on how the investigators defined the aim of an intervention since interventions often have multiple aims (e.g., strengthen parenting behaviour and promote child mental health) and the aim stated in an evaluation often depends on the research question published in a specific report. Only studies that included parents of children aged 2–10 years were included. We included peer-reviewed publications, as well as unpublished manuscripts, dissertations, and results published in trial registries. This systematic review is part of a larger set of systematic reviews with a range of effectiveness questions that was in part conducted for the development of the WHO Guidelines on Parenting interventions to prevent maltreatment and enhance parent–child relationships in children aged 0–17 years,^8^ and informed the development of recommendation 2.

For this research question, we included only trials that examined physical or emotional violence against children. Physical and emotional violence include any physical or verbal punishment or aggression, words or acts that cause harm, potential harm or threat of harm to a child. For inclusion, 50% of items of an instrument or sub-scale of an instrument needed to include physical or emotional violent behaviours. Full inclusion criteria can be found in the online supplement (Table S1).

### Data analysis

Three authors (SB, JJ, PL) independently extracted data for the included trials using a piloted extraction form. Extracted data included information on the publication, study setting/context; intervention characteristics; and on the study population. We assessed risk of bias of included studies using the Cochrane Risk of Bias Tool for RCTs.^24^ Certainty in the overall effect estimate was assessed using the Grading of Recommendations Assessment, Development and Evaluation (GRADE) approach.^25^

We calculated Cohen's d using the post sample size, means and standard deviations for intervention and control group. Where these data were not reported, we used relevant model statistics that were based preferably on intention-to-treat analyses. For model derived statistics or regression coefficients, we extracted information on covariates and adjustments wherever possible. Where trials included multiple arms, we extracted each intervention control comparison with reference to a common comparator. We contacted trial authors to obtain missing data for quantitative analyses. Robust variance estimation (RVE) was used to synthesise effect sizes using the robumeta package in STATA.^26^ RVE meta-analysis allows to include all effect sizes even when the nature of their dependence is unknown. We used a random effects meta-analysis model and assumed an intercorrelation of 0.8. Heterogeneity (I^2^) was calculated using the Q-value and degrees of freedom obtained from the RVE meta-analyses.

We grouped effect sizes by time point of assessment under three overarching time categories: immediate effects (up to 1 month after the intervention), 1–6 months follow-up effects, and 7–24 months follow-up effects. This approach was chosen based on our knowledge of the parenting intervention field and literature with most trials reporting immediate effects, some trials effects up to 6 months, and few trials reporting longer follow-up effects.^27^, ^28^, ^29^ The longest follow-up effect of included studies was 24 months (Fig. S5). Outcome measures at various time points were only included if the randomised design was still intact.

We ran RVE meta-analyses to calculate main effects for each time point group. Then, we ran a meta-regression including time of measurement as a moderator using RVE (as a continuous moderator in weeks, and as a dummy variable in time point categories: immediate vs 1–6 months & 7–24 months follow-up). Sensitivity analyses excluded any potential outliers. Publication bias was assessed visually using funnel plots. Due to the dependency of effect sizes, Egger's regression as well as the Trim and Fill method are at high risk for Type I error and not a recommended method when using robust variance estimation.^30^

### Pre-registration of review

The protocol of this systematic review was registered on PROSPERO (CRD42019141844).

### Ethical approval and informed consent

This study received ethical approval from the Department of Social Policy and Intervention at the University of Oxford. Due to the inclusion of only publicly available trial-level summary data, no additional informed consent was needed for this review.

### Role of the funding source

The funders of this study had no role in study design, data collection, data analysis, data interpretation, or writing of the Article. All authors had full access to the data in the study and had final responsibility for the decision to submit for publication.

## Results

We screened 20,860 abstracts with an interrater agreement of 95% (in 2019: 13,022; update in 2022: 7838; Fig. 1). A total of 346 trials were eligible for inclusion in this systematic review, but only 60 trials reported physical or emotional violence outcomes. Tables 1 and 2 show the characteristics of the 60 included trials. Studies were published between 1984 and 2022; two unpublished manuscripts were included. Interventions were evaluated in 23 countries across various income groups as defined by the World Bank. The majority of trials were implemented in high-income countries (78%, n = 47), and most interventions were homegrown in the trial country (72%, n = 43). The most represented country was the United States of America (47%, n = 28).

**Fig. 1:**
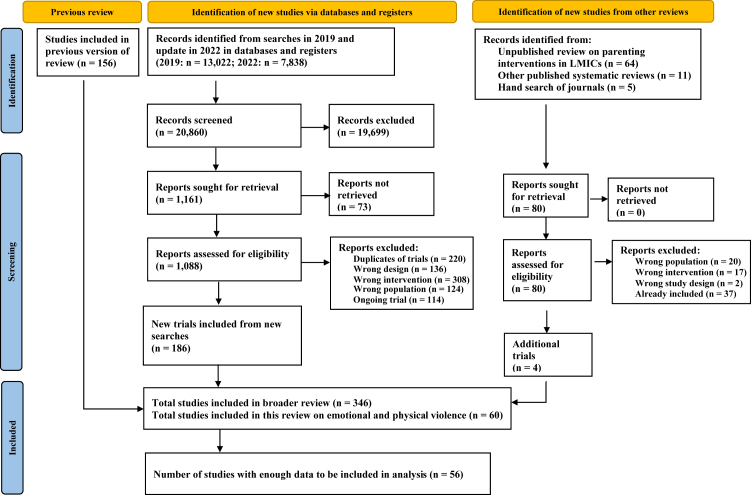
**Preferred reporting items for systematic reviews and meta-analyses (PRISMA) flow-chart**.

**Table 1:** Intervention and design characteristics of included studies (n = 60).

	Year	N	Country	Intervention	Control	Immediate effects	1–6 months effects	7–24 months effects	Outcome	% of violence items
Al-Hassan^a^^,^^31^	2011	337	Jordan	Better Parenting Programme	No treatment	0 weeks	–	–	Beating the child (*Unknown Questionnaire*)	100%
Arrubarrena^32^	2022	146	Spain	Incredible Years	Care as usual	–	26 weeks	52 weeks	Physical Punishment (Parenting Practice Inventory)	100%
Bailey^33^	2015	12	Australia	1-2-3 Magic Parenting Program	Wait-list	0 weeks	–	–	Hostility (Parenting Scale)	100%
Bradley^34^	2003	198	Canada	1-2-3 Magic Parenting Program	Wait-list	–	5 weeks	–	Hostility (Brief Symptom Inventory)	100%
Braet^35^	2009	64	Belgium	Parent Management Training	Wait-list	0 weeks	–	–	Harsh punishment (Ghent Parental Behavior Scale)	100%
Breitenstein^36^	2012	24	USA	Chicago Parent Program	Wait-list	0 weeks	24 weeks	48 weeks	Corporal punishment (Parenting Questionnaire)	100%
Breitenstein^37^	2016	79	USA	Chicago Parent Program	Minimal intervention	0 weeks	12 weeks	–	Corporal punishment (Parenting Questionnaire)	100%
Breitenstein^38^	2021	287	USA	Chicago Parent Program	Care as usual	–	12 & 26 weeks	52 weeks	Corporal punishment (Parenting Questionnaire)	100%
Chacko^39^	2018	126	USA	Fathers Supporting Success in Preschoolers	Wait-list	0 weeks		–	Corporal and verbal punishment (Parent Behavior Checklist)	70%
Cheng^40^	2004	281	USA	DARE To Be You	No treatment	0 weeks	–	52 weeks	Harsh/corporal punishment (Harsh Punishment Scale)	likely >50%
Day^41^	2018	183	Australia	Online Triple P	Wait-list	0 weeks	20 weeks	–	Hostility (Parenting Scale)	100%
DeGarmo^42^	2019	426	USA	Fathering through Change	Wait-list	–	12 weeks	–	Harsh discipline (Parenting Practice Inventory)	100%
Foskolos^43^	2014	124	Greece	Triple P	Minimal intervention	0 weeks	26 weeks	–	Hostility (Parenting Scale)	100%
Francis^44^	2021	223	Jamaica	Irie Homes Toolbox	Wait-list	–	12 weeks	–	Psychological aggression (Conflict Tactics Scale) Corporal punishment (Conflict Tactics Scale)	100%
Fung^45^	2014	137	USA	Early Pathways	Wait-list	0 weeks	–	–	Verbal and corporal punishment (Parent Behavior Checklist)	70%
Furlong^46^	Un-published	41	Ireland	ChARM	Wait-list	–	–	52 weeks	Abuse (total sibling, psychological target child)	100%
Gardner^47^	Un-published	120	Thailand	Parenting for Lifelong Health	Care as usual	–	12 weeks	–	Physical abuse (ISPCAN Screening Tool for use in Trials), Emotional abuse (ISCPAN Screening Tool for use in Trials)	100%
Gross^48^	2009	253	USA	Incredible Years	No treatment	0 weeks	–	–	Corporal punishment (Parenting Questionnaire)	100%
Guterman^49^	2013	138	USA	Parent Aide	Care as usual	–	26 weeks	–	Psychological aggression (Conflict Tactics Scale), Physical assault (Conflict Tacticts Scale), Hostility (Brief Symptom Inventory)	100%
Guo^50^	2016	81	China	Triple P	Wait-list	0 weeks	–	–	Corporal punishment (Alabama Parenting Questionnaire)	100%
Harris^51^	2015	199	USA	Early Pathways	Wait-list	0 weeks	–	–	Verbal and corporal punishment (Parent Behavior Checklist)	70%
Herbert^52^	2013	31	USA	Parenting Your Hyperactive Preschooler	Wait-list	0 weeks	–	–	Punitive reactions (Coping with Child Negative Emotions)	100%
Javier^53^	2016	28	USA	The Filipino Family Initiative	Wait-list	0 weeks	–	–	Physical punishment (Parenting Practice Inventory)	100%
Jouriles^54^	2001	36	USA	Project Support	Care as usual	–	–	50 weeks	Maternal aggression towards the child (Conflict Tactics Scale)	100%
Jouriles^55^	2009	66	USA	Project Support	Care as usual	0 weeks	–	–	Physical assault (Conflict Tactics Scale), Psychological aggression (Conflict Tactics Scale)	100%
Knox^56^	2013	149	USA	ACT Raising Safe kids	Care as usual	0 weeks	–	–	Psychological aggression (Conflict Tactics Scale)	100%
Lachman^57^	2017	68	South Africa	Parenting for Lifelong Health	Wait-list	0 weeks	–	–	Harsh parenting: physical assault and psychological aggression (Conflict Tactics Scale)	100%
Lachman^58^	2021	120	Philippines	Parenting for Lifelong Health	Care as usual	4 weeks	–	52 weeks	Physical abuse (ISPCAN Screening Tool for use in Trials), Emotional abuse (ISCPAN Screening Tool for use in Trials)	100% 100%
Leijten^59^	2017	154	Netherlands	Incredible Years	Wait-list	0 weeks	–	–	Physical punishment (Parenting Practice Inventory)	100%
Lessard^60^	2016	96	Canada	Incredible Years	Care as usual	0 weeks	–	–	Physical punishment (Parenting Practice Inventory)	100%
Lester^61^	2014	80	South Africa	Positive Parenting Skills Training Programme	Wait-list	0 weeks	–	–	Hostile parenting (Parent Behavior Inventory)	50%
Leung^62^	2015	111	Hong Kong	Parent–Child Interaction Therapy	Wait-list	0 weeks	–	–	Corporal punishment (Observation)	100%
Leung^63^	2017	64	Hong Kong	Parent–Child Interaction Therapy	Wait-list	0 weeks	–	–	Corporal punishment (Observation)	100%
Menting^64^	2014	133	Netherlands	Incredible Years	No treatment	0 weeks	–	–	Corporal punishment (Alabama Parenting Questionnaire)	100%
Miller-Heyl^65^	1998	796	USA	DARE To Be You	No treatment	–	–	40 & 92 weeks	Harsh punishment (Self-developed)	likely 100%
Nicholson^66^	2002	26	USA	STAR parenting programme	Wait-list	0 weeks	–	–	Harsh discipline (Parent Behavior Checklist)	70%
Nogueira^67^	2021	134	Portugal	Triple P	Care as usual	2 weeks	26 weeks	52 weeks	Hostility (Parenting Scale)	100%
Olivares^68^	1997	60	Spain	*unnamed*	Minimal intervention	4 weeks	–	52 weeks	Punishment non-verbal (Observation)	likely 100%
Oveisi^69^	2010	246	Iran	SOS! Help for parents	Care as usual	–	8 weeks	–	Child abuse (Conflict Tactics Scale)	100%
Peterson^70^	2002	119	USA	*unnamed*	No treatment	0 weeks	–	–	Harsh punishment (Self-developed)	100%
Portwood^71^	2011	271	USA	ACT Raising Safe Kids	Care as usual	0 weeks	12 weeks	–	Harsh discipline (Parent Behavior Checklist)	70%
Prinz^a^^,^^72^	2009	195,270	USA	Triple P	No treatment	–	–	260 weeks	Official report	100%
Pruett^73^	2019	284	USA	Supporting Father Involvement	Wait-list	–	8 weeks	–	Harsh/corporal punishment (Alabama Parenting Questionnaire)	100%
Rincón^74^	2018	332	Chile	Day by Day Program	Wait-list	–	5 weeks	–	Humiliating treatment (Harsh Discipline Practice List), Physical punishment (Harsh Discipline Practice List)	100%
Selby^a^^,^^75^	2021	129	UK	Embers the Dragon Intervention	Care as usual	0 weeks	–	–	Hostility (Parenting Scale)	100%
Self-Brown^76^	2018	99	USA	SafeCare	Minimal intervention	1 week	–	–	Corporal punishment (Conflict Tactics Scale), Psychological aggression (Conflict Tactics Scale)	100%
Silovsky^a^^,^^77^	2011	105	USA	SafeCare	Care as usual	–	–	96 weeks	Official report	100%
Sim^78^	2014	270	Liberia	Parents Make the Difference	Wait-list	4 weeks	–	–	Harsh discipline (MICS Child Discipline Module)	100%
Smith^79^	2010	60	USA	Role of a Father	Wait-list	0 weeks	–	–	Discipline: Harsh, punishing (Parent Behavior Checklist)	70%
Solís-Cámara^80^	2004	40	Mexico	Programa de Crianza Estandarizado	Wait-list	0 weeks	–	–	Negative verbal behaviour (Observation), Negative physical behaviour (Observation), Harsh Discipline (Parent Behavior Checklist)	100%
Solís-Cámara^81^	2015	60	Mexico	Programa de Crianza Estandarizado.	No treatment	0 weeks	–	–	Negative verbal behaviour (Observation), Negative physical behaviour (Observation), Harsh Discipline (Parent Behavior Checklist)	70%
Sourander^82^	2016	464	Finland	Strongest Families Smart Website	Minimal intervention	–	12 weeks	40 & 92 weeks	Hostility (Parenting Scale)	100%
Spaccarelli^83^	1992	37	USA	Incredible Years	Wait-list	0 weeks	–	–	Punitiveness, coercive techniques (Parent Behavior Inventory)	50%
Villodas^84^	2021	55	USA	Parent–Child Interaction Therapy	Care as usual	0 weeks	–	–	Punitive punishment (Alabama Parenting Questionnaire)	100%
Ward^85^	2020	296	South Africa	Parenting for Lifelong Health	Care as usual	0 weeks	–	52 weeks	Physical discipline (ISPCAN Screening Tool for use in Trials), Psychological discipline (ISPCAN Screening Tool for use in Trials)	100%
Webster–Stratton^86^	1984	25	USA	Incredible Years	Wait-list	0 weeks	–	–	Spanking (Parent Daily Report)	100%
Webster–Stratton^87^	1990	50	USA	Incredible Years	Wait-list	4 weeks	–	–	Spanking (Parent Daily Report)	100%
Wolfe^88^	1988	53	Canada	Parent training	Care as usual	0 weeks	12 weeks	–	Physical negative (Observation)	likely 90%
Yao^89^	2022	30	Japan	Behaviour parent training	Wait-list	0 weeks	–	–	Yelling (Parenting Scale) Spanking (Parenting Scale)	100% 100%
Zahra^90^	2014	60	Iran	*unnamed*	Care as usual	0 weeks	–	–	Emotional abuse (Conflict Tactics Scale), Physical aggression (Conflict Tactics Scale)	100%

a Not included in meta-analysis.

**Table 2:** Participant characteristics of included studies (n = 60).

	Year	N	Intervention	Prevention level	Child gender (boys)	Child mean age	Child age range
Al-Hassan^a^^,^^31^	2011	337	Better Parenting Programme	Universal	–	–	0–8 years
Arrubarrena^32^	2022	146	Incredible Years	Treatment	66%	6.6	4–8 years
Bailey^33^	2015	22	1-2-3 Magic Parenting Program	Universal	75%	8.50	6–12 years
Bradley^34^	2003	198	1-2-3 Magic Parenting Program	Selective	61%	3.75	3–4 years
Braet^35^	2009	64	Parent Management Training	Selective	64%	5.58	4–7 years
Breitenstein^36^	2012	24	Chicago Parent Program	Universal	54%	2.81	2–4 years
Breitenstein^37^	2016	79	Chicago Parent Program	Universal	43%	–	2–5 years
Breitenstein^38^	2021	287	Chicago Parent Program	Universal	–	2.2	2–5 years
Chacko^39^	2018	126	Fathers Supporting Success in Preschoolers	Selective	68%	4.59	–
Cheng^40^	2004	281	DARE To Be You	Universal	–	–	2–5 years
Day^41^	2018	183	Online Triple P	Selective	47%	3.50	1–8 years
DeGarmo^42^	2019	426	Fathering through Change	Universal	56%	7.88	4–12 years
Foskolos^43^	2014	124	Triple P	Universal	53%	–	2–12 years
Francis^44^	2021	223	Irie Homes Toolbox	Universal	51%	4.04	–
Fung^45^	2014	137	Early Pathways	Selective	73%	3.90	0–6 years
Furlong^46^	Un-published	41	ChARM	Treatment	61%	6.60	–
Gardner^47^	Un-published	120	Parenting for Lifelong Health	Selective	61%	5.22	2–9 years
Gross^48^	2009	253	Incredible Years	Universal	56%	2.91	2–4 years
Guterman^49^	2013	138	Parent Aide	Indicated	50%	–	0–12 years
Guo^50^	2016	81	Triple P	Universal		8.05	–
Harris^51^	2015	199	Early Pathways	Selective	70%	2.88	1–5 years
Herbert^52^	2013	31	Parenting Your Hyperactive Preschooler	Selective	74%	4.50	3–6 years
Javier^53^	2016	28	The Filipino Family Initiative	Selective	45%	8.45	6–12 years
Jouriles^54^	2001	36	Project Support	Selective	50%	5.67	4–9 years
Jouriles^55^	2009	66	Project Support	Selective	72%	–	4–9 years
Knox^56^	2013	149	ACT Raising Safe kids	Selective	59%	3.35	1–8 years
Lachman^57^	2017	68	Parenting for Lifelong Health	Selective	51%	5.40	3–8 years
Lachman^58^	2021	120	Parenting for Lifelong Health	Indicated	47%	3.80	2–6 years
Leijten^59^	2017	154	Incredible Years	Selective	62%	5.59	3–8 years
Lessard^60^	2016	96	Incredible Years	Selective	84%	8.20	6–9 years
Lester^61^	2014	80	Positive Parenting Skills Training Programme	Universal	56%	8.36	5–12 years
Leung^62^	2015	111	Parent–Child Interaction Therapy	Selective	74%	4.50	2–7 years
Leung^63^	2017	64	Parent–Child Interaction Therapy	Selective	83%	5.50	2–7 years
Menting^64^	2014	133	Incredible Years	Selective	49%	6.40	2–10 years
Miller-Heyl^65^	1998	796	DARE To Be You	Selective	–	3.15	2–5 years
Nicholson^66^	2002	26	STAR parenting programme	Indicated	54%	–	1–5 years
Nogueira^67^	2021	134	Triple P	Selective	12%	7.13	3–12 years
Olivares^68^	1997	60	*unnamed*	Universal	–	7.50	7–9 years
Oveisi^69^	2010	246	SOS! Help for parents	Universal	51%	4.53	2–6 years
Peterson^70^	2002	119	*unnamed*	Indicated	62%	3.00	2–4 years
Portwood^71^	2011	271	ACT Raising Safe Kids	Universal	–	–	0–7 years
Prinz^a^^,^^72^	2009	195,270	Triple P	Universal	–	–	0–8 years
Pruett^73^	2019	284	Supporting Father Involvement	Treatment	–	2.90	0–12 years
Rincón^74^	2018	332	Day by Day Program	Universal	53%	3.80	3–5 years
Selby^a^^,^^75^	2021	129	Embers the Dragon Intervention	Universal	47%	4.96	2–7 years
Self-Brown^76^	2018	99	SafeCare	Selective	65%	3.30	2–5 years
Silovsky^a^^,^^77^	2011	105	SafeCare	Selective	–	–	?-5 years
Sim^78^	2014	270	Parents Make the Difference	Selective	47%	5.16	3–7 years
Smith^79^	2010	60	Role of a Father	Selective		>50%: 1–9 years	1–18 years
Solís-Cámara^80^	2004	40	Programa de Crianza Estandarizado	Selective	60%	3.70	3–5 years
Solís-Cámara^81^	2015	60	Programa de Crianza Estandarizado.	Universal	63%	3.68	3–5 years
Sourander^82^	2016	464	Strongest Families Smart Website	Selective	62%	4	4 years
Spaccarelli^83^	1992	37	Incredible Years	Universal	57%	6.20	–
Villodas^84^	2021	55	Parent–Child Interaction Therapy	Selective	62%	4.78	2–7 years
Ward^85^	2020	296	Parenting for Lifelong Health	Selective	53%	–	2–9 years
Webster–Stratton^86^	1984	25	Incredible Years	Indicated	71%	4.80	3–8 years
Webster–Stratton^87^	1990	50	Incredible Years	Selective	79%	5.00	3–8 years
Wolfe^88^	1988	53	Parent training	Indicated	–	2.04	0–5 years
Yao^89^	2022	30	Behaviour parent training	Selective	–	–	6–12 years
Zahra^90^	2014	60	*unnamed*	Treatment	–	5	5 years

a Not included in meta-analysis.

Most interventions targeted families at risk for maltreatment (selective prevention; 50%, n = 30), followed by universal prevention (33%, n = 20), while only a few included families based on their levels of child maltreatment such as presence of corporal punishment in the family (indicated prevention, 10%, n = 6), or previous referral to social protection services based on a history of maltreatment (treatment, 7%, n = 4). Sample size ranged from 12 to 796 families. Most programmes were delivered in group format (58%, n = 35) and compared to a wait-list control group (48%, n = 29), followed by care as usual (28%, n = 17), no treatment (15%, n = 9), and minimal intervention (8%, n = 5). The mean age of included parents at baseline was 33.43 (SD = 4.94) and of their children was 4.97 years (SD = 1.78). The most used instruments to measure physical and emotional violence were the Conflict Tactics Scale Parent–Child Version, the Hostility scale of the Parenting Scale, and the Corporal Punishment scale of the Alabama Parenting Questionnaire (Table 1). Most effect sizes were based on parent self-report (91%, k = 97). The mean number of timepoints was 1.34 (SD = 0.58), with most trials only including immediate post-test effects (n = 33). The longest follow-up measurement included in the meta-analyses was 92 weeks (n = 1).

**Fig. 2:**
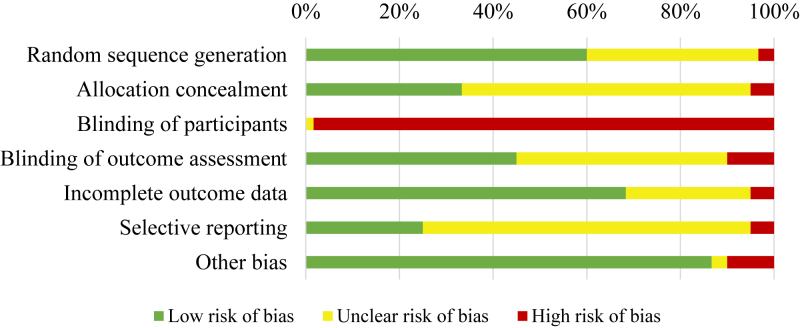
**Summary of risk of bias assessment across studies (n = 60)**.

Risk of bias was concerning for blinding of outcome assessors because the majority of data was self-reported by parents which is generally at high risk of bias (Fig. 2). We observed poor reporting of allocation concealment and blinding of outcome assessors, and low rates of registered protocols (and consequently unclear risk of selective reporting). For random sequence generation, incomplete outcome data, and other bias, the majority of studies received low risk of bias ratings.

**Fig. 3:**
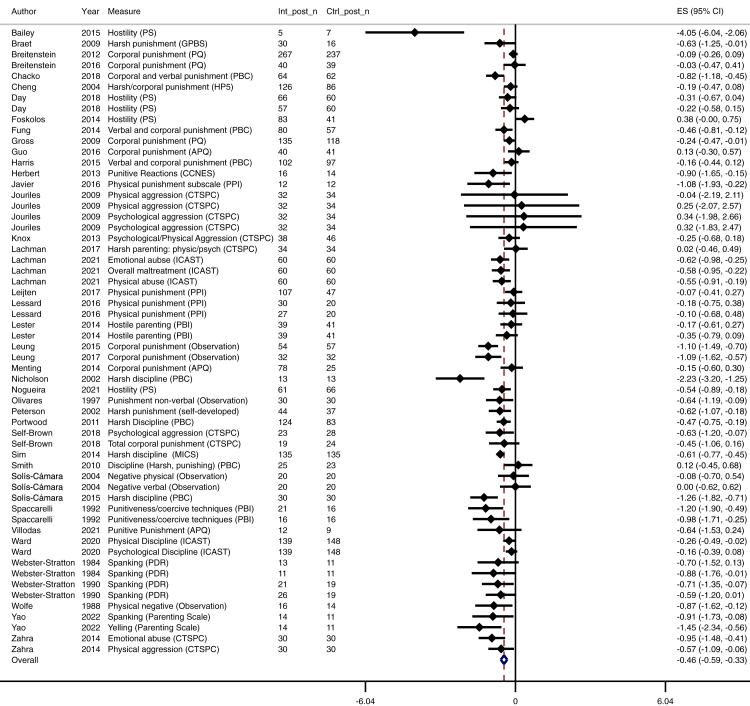
**Forest plot for the immediate effect of parenting interventions on physical and emotional violence after the intervention (0–4 weeks after intervention).** ES = Effect size, CI = Confidence interval, Int_post_n = Sample size for intervention group at post-test, Ctrl_post_n = Sample size for control group at post-test.

**Fig. 4:**
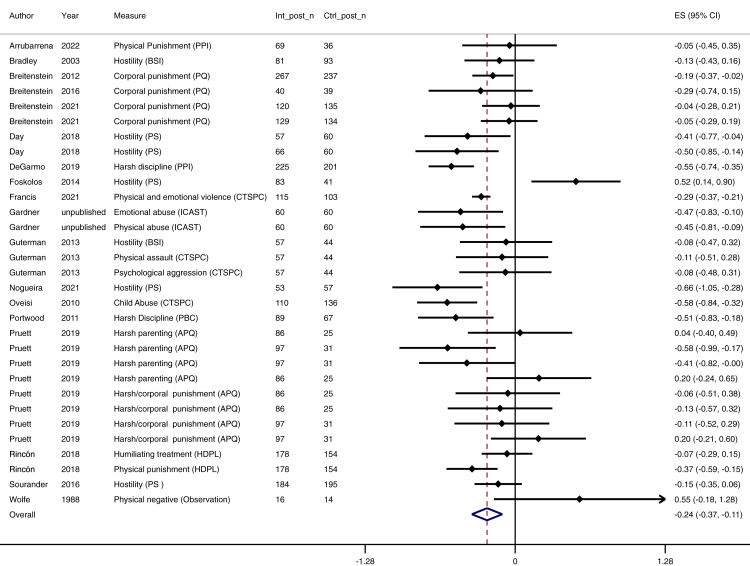
**Forest plot for the effect at 1–6 months follow-up of parenting interventions on physical and emotional violence.** ES = Effect size, CI = Confidence interval, Int_post_n = Sample size for intervention group at post-test, Ctrl_post_n = Sample size for control group at post-test.

**Fig. 5:**
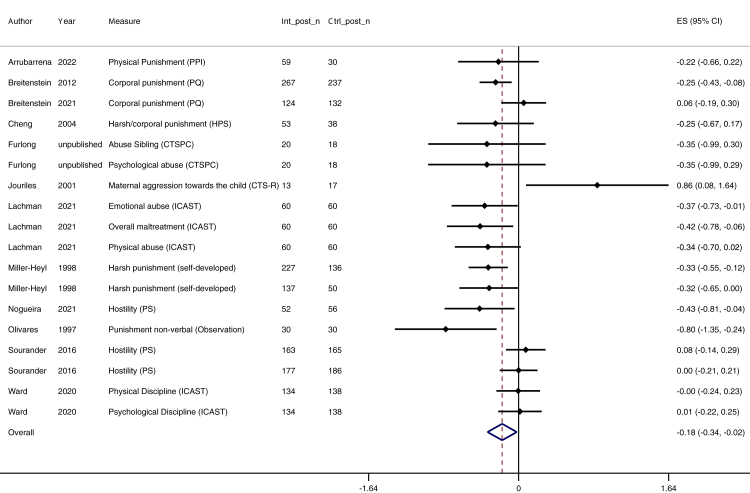
**Forest plot for the effect at 7–24 months follow-up of parenting interventions on physical and emotional violence.** ES = Effect size, CI = Confidence interval, Int_post_n = Sample size for intervention group at post-test, Ctrl_post_n = Sample size for control group at post-test.

We found an overall small effect of parenting interventions for reducing physical and emotional violence (n = 56, k = 107; *d* = −0.37; 95% CI [–0.47, −0.27]) in a heterogeneous set of effect sizes (*I*^2^ = 76%). The number of trials with longer follow up was relatively small, and although effects were maintained over time, the effect sizes appeared to lessen over time (Table 3): immediately after the intervention (up to 1 month) *d* = −0.46 (n = 42, k = 58; 95% CI [–0.59, −0.33], *I*^2^ = 76%; moderate certainty; Fig. 3), 1–6 months follow-up *d* = −0.24 (n = 18, k = 31; 95% CI [–0.37, −0.11], *I*^2^ = 73%; low certainty; Fig. 4), and 7–24 months follow-up *d* = −0.18 (n = 12, k = 18; *9*5% CI [–0.34, −0.02], *I*^2^ = 61%; low certainty; Fig. 5). Publication bias was suspected for 7–24 months follow-up data, with an overrepresentation of larger trials reporting beneficial longer-term effects (see online supplement, Figs. S1–S3). Main effect results are summarised in Table 3.

**Table 3:** Meta-analytic results for all timepoint categories.

Time point	No. of trials	No. of effect sizes	Effect size (Cohen's d)	N intervention group	N control group	Confidence interval of effect size	Hetero-geneity (I^2^)	GRADE certainty of evidence	Publication bias
Across all time points	56	107	−0.37	3992	3478	−0.47, −0.27	76%	⨁⨁⨁◯ moderate	Not detected
Immediate	42	58	−0.46	2388	2062	−0.59, −0.33	76%	⨁⨁⨁◯ moderate	Not detected
1–6 months	18	31	−0.24	1901	1703	−0.37, −0.11	73%	⨁⨁◯◯ low	Not detected
7–24 months	12	18	−0.18	1264	1135	−0.34, −0.02	61%	⨁⨁◯◯ low	Detected

The main effects for each time point suggest that effects decrease over time. Meta-regression results with the number of weeks after the intervention as a continuous moderator revealed a significant moderation effect (β = 0.005; 95% CI [0.00, 0.00], τ^2^ = 0.06), and tested categorically, there was a significant difference in effect estimates between immediate (*d* = −0.46), and 1–6 and 7–24 months follow-up effects (β = 0.24; 95% CI [0.07, 0.40]).

We conducted various post-hoc robustness-checks on our findings. First, we tested whether the decrease in effects over time could be explained by publication bias with trials with lower initial intervention success (smaller Cohen's d at post-test) not publishing follow-up effects. While we were unable to exclude this possibility, we tested whether trials with follow-up effects reported higher magnitude of effect at post-test compared to trials without follow-up data using meta-regression analyses on the mean Cohen's d at post-test for both groups of trials. This was not the case (β = 0.26; 95% CI [−0.01, 0.52], τ^2^ = 0.09). Second, we tested whether our decision to include effect sizes from both instruments developed to measure harsh parenting and instruments developed to measure child maltreatment instruments impacted the results. For this, we reran our analyses, first, including only validated and explicit child maltreatment instruments, and, second, varying the cut-off for the number of items that measure physical or emotional violence that instruments had to include in order to be included in our meta-analysis (25%, 75%, 100%). For these sensitivity analyses, we included all time points, since analyses would otherwise fail to produce a reliable estimate due to a small number of trials included. We found a small effect for parenting interventions on physical and emotional violence for maltreatment validated instruments (ICAST & CTSPC) across all time points (n = 12, k = 32; *d* = −0.24; 95% CI [–0.47, −0.02]; *I*^*2*^ = 69%). Consequently, the effect holds when using a more conservative inclusion criterion for instruments. We found moderate effects for parenting interventions on physical and emotional violence when using various cut-offs for the number of items measuring physical or emotional violence i) 25% (n = 204, k = 108; *d* = −0.38; 95% CI [–0.46, −0.31]; *I*^*2*^ = 74%), ii) 75% (n = 45, k = 92; *d* = −0.32; 95% CI [–0.42, −0.22]; *I*^*2*^ = 73%), iii) 100% (n = 44, k = 90; *d* = −0.32; 95% CI [–0.43, −0.22]; *I*^*2*^ = 73%). This indicates that the overall main effect is robust and present even with wider or stricter instrument inclusion criteria (i.e., including only instruments that solely measured physical and emotional violence).

## Discussion

With parenting interventions being distributed globally, it is vital to understand whether they effectively and sustainably reduce physical and emotional violence against children. This study found that parenting interventions based on social learning theory effectively reduce physical and emotional violent parenting behaviours. Intervention effects were maintained but smaller over time, even though the evidence base was limited to intervention effects up to 24 months.

The small overall effect of *d* = −0.37 (based on 7470 families) indicates that Cohen's d of −0.37 would mean that 65% of the intervention group will show less physical and emotional violence than the control group (Cohen's U3), whereas still 35% of participants are expected to show similar scores as the control group.^91^ However, keeping in mind that parenting interventions are gradually being scaled up, as a consequence more families would have access to interventions which strongly increases the number of children that can be protected from violence at home. In addition to this, evidence for their effects, including effects over time, is more robust than that of other interventions that aim to reduce violence against children (such as changing norms and life skills trainings).^6^

We found that effects got smaller over time. Coercive cycles suggest that patterns of parent-child interaction become more entrenched over time and thus harder to change. Our findings suggest that change in these manifested parenting patterns through parenting interventions is possible, and that effects from interventions possibly maintain over time, albeit over time parents may partially apply previous parenting patterns. Based on the number of relatively few trials examining effects beyond 1 year post intervention, conclusions on longer-term effects remain undrawn. Findings from previous meta-analyses on the effects of parenting interventions over time are mixed. While most parenting reviews could not examine effects at follow-up due to a lack of studies examining follow-up effects,^17^^,^^27^^,^^28^ exploratory meta-analyses suggest sustained effects on parenting.^13^^,^^18^ Turning towards the broader literature, two reviews examined the effects of parenting intervention for young children (under 3 years of age) over time. While one review with a broader scope in outcomes found fading out effects,^92^ a review focusing on child abuse and neglect found no effects at follow-up. Our study adds to this body of evidence by suggesting that parenting programme effects maintain but get smaller over time, although the number of studies is too small to draw more firm conclusions.

We were unable to test whether parenting interventions effects maintain or further diminish beyond 2 years post-intervention. That the effect sizes at 1–6 months follow-up (*d* = −0.24) and 7–24 months follow-up (*d* = −0.18) seemed similar suggest that effects may decrease in the initial weeks and months after the intervention ends, but effects can still be observed in studies reporting follow-up data. However, analyses within trials that report post-test and follow-up data are urgently needed to fully exclude publication bias, and longer follow-up studies are needed to confirm our findings.

Our findings suggest that a key challenge for the field is to understand how effects can be sustained over time. For example, booster sessions may sustain initial effects, or at least weaken or delay potential fade-out effects. Another option would be interventions that are briefer, but ongoing, such as the Family Check-Up system where families are seen yearly to assess family strengths and challenges and offer tailored additional support based on this assessment.^93^ However, future studies should consider these additional costs related to sustaining effects and re-evaluate the cost-effectiveness of parenting interventions to reduce violence against children. We know from other public health behavioural interventions that sustained behaviour change is challenging (e.g., obesity interventions,^94^ smoking cessation^95^). Various theories have been developed to understand threats to long-term behaviour adherence.^96^, ^97^, ^98^ For the parenting intervention field, more research is urgently needed to identify why some parents struggle to maintain their new parenting skills. Possible explanations could be related to change in parents’ motivation, capability, or opportunities over time (COM-B model^99^), changes in the context of the behaviour such as children moving into new developmental stages with new challenging behaviors,^97^ or possible friction in the environment by only one parent applying the new behavior.^98^

Several limitations of our review merit attention. First, our outcomes included both parent self-report and direct observational data, but most effect sizes (91%, n = 97) were based on self-report of parents. Albeit self-report of maltreatment yields a more reliable estimate than official reports, self-report data is at risk for social desirability bias, particularly given that blinding of participants was not possible in the included trials.^100^ Results of any evaluation of interventions to reduce child maltreatment should be seen in the light of the restrictions of our measures to validly assess maltreatment. Second, we included only interventions based on social learning theory. This allowed us to reduce heterogeneity between trials and included the commonest and most established parenting interventions (e.g., Triple P, Incredible Years, Parenting for Lifelong Health, Parent–Child Interaction Therapy, and Parent Management Training—Oregon model), but limited the generalisability of our findings to this specific type of interventions. While multiple evaluation studies of the same intervention brand were often included in the same meta-analysis, no specific intervention dominated any of the meta-analyses. Third, we observed high heterogeneity in effect sizes despite relative homogeneity in intervention type and age group. This heterogeneity may reflect differential effects based on the study population, intervention setting, delivery agents, etc. Since moderation analyses in meta-analyses are generally underpowered,^101^ individual participant data meta-analysis that can study differential intervention effects at the individual family level is needed to unpack which factors impact intervention effects on physical and emotional violence. Fourth, only a few trials provided immediate post-test and follow-up data. Thus, we were unable to compare effects over time within trials. Fifth, this review could not estimate the effects of parenting interventions for physical and emotional violence separately since most studies used measurements that merge physical and emotional violence. Future trials should separate out physical and emotional violence.

Despite these limitations, our study answers to a timely call by international organisations to examine effects over time of parenting interventions to reduce violence against children. Methodological rigor in terms of systematic literature search (published and unpublished work in multiple languages), inclusion (i.e., only randomised controlled trials; measures of violence regardless of whether they are labelled as such; interventions with the same theory and similar components), and analyses (robust variance estimation including all eligible effect sizes and sensitivity analyses ruling out alternative explanations) enhance the credibility of our findings.

In conclusion, parenting interventions based on social learning theory can successfully reduce physical and emotional violence perpetrated by parents and caregivers, even though effects are reduced at follow-up. With global policy interest and scale up of interventions, research is urgently needed to identify how effects can be better sustained over time.

## Contributors

SB coordinated the review of all articles, conducted the searches, screened studies from other reviews for inclusion, extracted data, ran the analyses, and wrote the paper. SB, PL, and FG were responsible for the conception, design, interpretation of results and overall oversight of the review. FG led an earlier version of the review. PL contributed to the writing of the paper and assisted in the data extraction. JJ collaborated on study screening, data extraction, and quality appraisal. GJMT supported the statistical analyses. All authors critically revised the Article for important intellectual content and approved the final version. SB, JJ, and PL accessed and verified the data. All authors had full access to the data in the study and had final responsibility for the decision to submit for publication.

## Data sharing statement

The review protocol is publicly available on PROSPERO and an extended version on Open Science Framework. Extraction data sheets will be made publicly available upon reasonable request to the lead author.

## Declaration of interests

PL and FG led trials that were included in the dataset. FG is a co-developer of a non-profit parenting programme with WHO, Parenting for Lifelong Health. SB and FG were members of the WHO Guideline Development Group on parenting and child maltreatment; SB and FG were members of the WHO Guideline Evidence Synthesis Team; SB wrote the WHO Guidelines on parenting interventions to prevent maltreatment and enhance parent–child relationships in children aged 0–17 years. JJ and GJMT declare that there is no conflict of interest.

## References

[1] UNICEF (2014).

[2] Hillis S., Mercy J., Amobi A., Kress H. (2016). Global prevalence of past-year violence against children: a systematic review and minimum estimates. Pediatrics.

[3] Marmor A., Cohen N., Katz C. (2021). Child maltreatment during COVID-19: key conclusions and future directions based on a systematic literature review. Trauma Violence Abuse.

[4] UN (1989). Convention on the rights of the child. https://treaties.un.org/doc/Treaties/1990/09/19900902%2003-14%20AM/Ch_IV_11p.pdf.

[5] UN (2016). Sustainable development goals. http://www.un.org/sustainabledevelopment.

[6] WHO (2016).

[7] Gilbert R., Fluke J., O'Donnell M. (2012). Child maltreatment: variation in trends and policies in six developed countries. Lancet.

[8] WHO (2023).

[9] Bandura A., McClelland D. (1977).

[10] Patterson G.R. (1982).

[11] Casillas K.L., Fauchier A., Derkash B.T., Garrido E.F. (2016). Implementation of evidence-based home visiting programs aimed at reducing child maltreatment: a meta-analytic review. Child Abuse Negl.

[12] Chen M., Chan K.L. (2016). Effects of parenting programs on child maltreatment prevention: a meta-analysis. Trauma Violence Abuse.

[13] Gubbels J., van der Put C.E., Assink M. (2019). The effectiveness of parent training programs for child maltreatment and their components: a meta-analysis. Int J Environ Res Public Health.

[14] Kennedy S.C., Kim J.S., Tripodi S.J., Brown S.M., Gowdy G. (2016). Does parent–child interaction therapy reduce future physical abuse? A meta-analysis. Res Soc Work Pract.

[15] McCoy A., Melendez-Torres G.J., Gardner F. (2020). Parenting interventions to prevent violence against children in low-and middle-income countries in East and Southeast Asia: a systematic review and multi-level meta-analysis. Child Abuse Negl.

[16] Van der Put C.E., Assink M., Gubbels J., Boekhout van Solinge N.F. (2018). Identifying effective components of child maltreatment interventions: a meta-analysis. Clin Child Fam Psychol Rev.

[17] Vlahovicova K., Melendez-Torres G.J., Leijten P., Knerr W., Gardner F. (2017). Parenting programs for the prevention of child physical abuse recurrence: a systematic review and meta-analysis. Clin Child Fam Psychol Rev.

[18] Euser S., Alink L.R., Stoltenborgh M., Bakermans-Kranenburg M.J., van IJzendoorn M.H. (2015). A gloomy picture: a meta-analysis of randomized controlled trials reveals disappointing effectiveness of programs aiming at preventing child maltreatment. BMC Public Health.

[19] (2016). Global partnership and fund to end violence against children.

[20] Backhaus S., Leijten P., Meinck F., Gardner F. (2022). Different instrument, same content? A systematic comparison of child maltreatment and harsh parenting instruments. Trauma Violence Abuse.

[21] Page M.J., Moher D., Bossuyt P.M. (2021). PRISMA 2020 explanation and elaboration: updated guidance and exemplars for reporting systematic reviews. BMJ.

[22] Higgins J.P.T., Thomas J., Chandler J. (2022). Cochrane handbook for systematic reviews of interventions version 6.3 (updated February 2022).

[23] Leijten P., Melendez-Torres G.J., Knerr W., Gardner F. (2016). Transported versus homegrown parenting interventions for reducing disruptive child behavior: a multilevel meta-regression study. J Am Acad Child Adolesc Psychiatry.

[24] Higgins J.P., Altman D.G., Gøtzsche P.C. (2011). The Cochrane Collaboration's tool for assessing risk of bias in randomised trials. BMJ.

[25] Guyatt G.H., Oxman A.D., Schünemann H.J., Tugwell P., Knottnerus A. (2011). GRADE guidelines: a new series of articles in the journal of clinical epidemiology. J Clin Epidemiol.

[26] Tanner-Smith E.E., Tipton E., Polanin J.R. (2016). Handling complex meta-analytic data structures using robust variance estimates: a tutorial in R. J Dev Life Course Criminol.

[27] Solís-Cordero K., Duarte L.S., Fujimori E. (2022). Effectiveness of remotely delivered parenting programs on caregiver-child interaction and child development: a systematic review. J Child Fam Stud.

[28] Coore Desai C., Reece J.A., Shakespeare-Pellington S. (2017). The prevention of violence in childhood through parenting programmes: a global review. Psychol Health Med.

[29] Pinquart M., Teubert D. (2010). Effects of parenting education with expectant and new parents: a meta-analysis. J Fam Psychol.

[30] Rodgers M.A., Pustejovsky J.E. (2021). Evaluating meta-analytic methods to detect selective reporting in the presence of dependent effect sizes. Psychol Methods.

[31] Al-Hassan S.M., Lansford J.E. (2011). Evaluation of the better parenting programme in Jordan. Early Child Dev Care.

[32] Arruabarrena I., De Paúl J., Rivas G.R., Cañas M. (2022). The incredible years parenting and child treatment programs: a randomized controlled trial in a child welfare setting in Spain. Psychosoc Interv.

[33] Bailey E.L., Van Der Zwan R., Phelan T.W., Brooks A. (2015). Keeping it going: evidence of long-term improvements after implementation of the 1-2-3 magic parenting program. Child Fam Behav Ther.

[34] Bradley S.J., Jadaa D.A., Brody J. (2003). Brief psychoeducational parenting program: an evaluation and 1-year follow-up. J Am Acad Child Adolesc Psychiatry.

[35] Braet C., Meerschaert T., Merlevede E., Bosmans G., Van Leeuwen K., De Mey W. (2009). Prevention of antisocial behaviour: evaluation of an early intervention programme. Eur J Dev Psychol.

[36] Breitenstein S.M., Gross D., Fogg L. (2012). The Chicago parent program: comparing 1-year outcomes for African American and Latino parents of young children. Res Nurs Health.

[37] Breitenstein S.M., Fogg L., Ocampo E.V., Acosta D.I., Gross D. (2016). Parent use and efficacy of a self-administered, tablet-based parent training intervention: a randomized controlled trial. JMIR Mhealth Uhealth.

[38] Breitenstein S.M., Fehrenbacher C., Holod A.F., Schoeny M.E. (2021). A randomized trial of digitally delivered, self-administered parent training in primary care: effects on parenting and child behavior. J Pediatr.

[39] Chacko A., Fabiano G.A., Doctoroff G.L., Fortson B. (2018). Engaging fathers in effective parenting for preschool children using shared book reading: a randomized controlled trial. J Clin Child Adolesc Psychol.

[40] Cheng S.H. (2004).

[41] Day J.J., Sanders M.R. (2018). Do parents benefit from help when completing a self-guided parenting program online? A randomized controlled trial comparing Triple P Online with and without telephone support. Behav Ther.

[42] DeGarmo D.S., Jones J.A. (2019). Fathering Through Change (FTC) intervention for single fathers: preventing coercive parenting and child problem behaviors. Dev Psychopathol.

[43] Foskolos K. (2014).

[44] Francis T., Baker-Henningham H. (2021). The irie homes toolbox: a cluster randomized controlled trial of an early childhood parenting program to prevent violence against children in Jamaica. Child Youth Serv Rev.

[45] Fung M.P., Fox R.A. (2014). The culturally-adapted early pathways program for young Latino children in poverty: a randomized controlled trial. J Lat Psychol.

[46] ∗ Unpublished data; Furlong M, Stokes A, McGilloway S, et al. Examining the effectiveness of a wraparound-inspired intervention for parents with children at risk of child maltreatment: outcomes from a multi-centre exploratory randomised controlled trial.

[47] ∗ Unpublished data; Gardner F, McCoy A, Lachman JM, Melendez-Torres GJ, Tapanya S, Loupha S. Randomised trial of a parenting intervention in the Thai public health system for reducing violence against children.

[48] Gross D., Garvey C., Julion W., Fogg L., Tucker S., Mokros H. (2009). Efficacy of the Chicago parent program with low-income African American and Latino parents of young children. Prev Sci.

[49] Guterman N.B., Tabone J.K., Bryan G.M., Taylor C.A., Napoleon-Hanger C., Banman A. (2013). Examining the effectiveness of home-based parent aide services to reduce risk for physical child abuse and neglect: six-month findings from a randomized clinical trial. Child Abuse Negl.

[50] Guo M., Morawska A., Sanders M.R. (2016). A randomized controlled trial of group Triple P with Chinese parents in Mainland China. Behav Modif.

[51] Harris S.E., Fox R.A., Love J.R. (2015). Early pathways therapy for young children in poverty: a randomized controlled trial. Couns Outcome Res Eval.

[52] Herbert S.D. (2013).

[53] Javier J.R., Coffey D.M., Schrager S.M., Palinkas L.A., Miranda J. (2016). Parenting intervention for prevention of behavioral problems in elementary school-age Filipino-American children: a pilot study in churches. J Dev Behav Pediatr.

[54] Jouriles E.N., McDonald R., Spiller L. (2001). Reducing conduct problems among children of battered women. J Consult Clin Psychol.

[55] Jouriles E.N., McDonald R., Rosenfield D., Stephens N., Corbitt-Shindler D., Miller P.C. (2009). Reducing conduct problems among children exposed to intimate partner violence: a randomized clinical trial examining effects of project support. J Consult Clin Psychol.

[56] Knox M., Burkhart K., Cromly A. (2013). Supporting positive parenting in community health centers: the ACT raising safe kids program. J Community Psychol.

[57] Lachman J.M., Cluver L., Ward C.L. (2017). Randomized controlled trial of a parenting program to reduce the risk of child maltreatment in South Africa. Child Abuse Negl.

[58] Lachman J.M., Alampay L.P., Jocson R.M. (2021). Effectiveness of a parenting programme to reduce violence in a cash transfer system in the Philippines: RCT with follow-up. Lancet Reg Health West Pac.

[59] Leijten P., Raaijmakers M.A., Orobio de Castro B., van den Ban E., Matthys W. (2017). Effectiveness of the incredible years parenting program for families with socioeconomically disadvantaged and ethnic minority backgrounds. J Clin Child Adolesc Psychol.

[60] Lessard J., Normandeau S., Robaey P. (2016). Effects of the incredible years program in families of children with ADHD. J Child Fam Stud.

[61] Lester S.N. (2014).

[62] Leung C., Tsang S., Sin T.C., Choi S.Y. (2015). The efficacy of parent–child interaction therapy with Chinese families: randomized controlled trial. Res Soc Work Pract.

[63] Leung C., Tsang S., Ng G.S., Choi S.Y. (2017). Efficacy of parent–child interaction therapy with Chinese ADHD children: randomized controlled trial. Res Soc Work Pract.

[64] Menting A.T., de Castro B.O., Wijngaards-de Meij L.D., Matthys W. (2014). A trial of parent training for mothers being released from incarceration and their children. J Clin Child Adolesc Psychol.

[65] Miller-Heyl J., MacPhee D., Fritz J.J. (1998). DARE to be you: a family-support, early prevention program. J Prim Prev.

[66] Nicholson B., Anderson M., Fox R., Brenner V. (2002). One family at a time: a prevention program for at-risk parents. J Couns Dev.

[67] Nogueira S., Abreu-Lima I., Canário C., Cruz O. (2021). Group Triple P – a randomized controlled trial with low-income mothers. Child Youth Serv Rev.

[68] Olivares J., Rosa A.I., López L.J. (1997). El papel del vídeo en el entrenamiento a madres: un estudio comparativo. Psicología Conductal.

[69] Oveisi S., Ardabili H.E., Dadds M.R. (2010). Primary prevention of parent-child conflict and abuse in Iranian mothers: a randomized-controlled trial. Child Abuse Negl.

[70] Peterson L., Tremblay G., Ewigman B., Popkey C. (2002). The parental daily diary: a sensitive measure of the process of change in a child maltreatment prevention program. Behav Modif.

[71] Portwood S.G., Lambert R.G., Abrams L.P., Nelson E.B. (2011). An evaluation of the adults and children together (ACT) against violence parents raising safe kids program. J Prim Prev.

[72] Prinz R.J., Sanders M.R., Shapiro C.J., Whitaker D.J., Lutzker J.R. (2009). Population-based prevention of child maltreatment: the US Triple P system population trial. Prev Sci.

[73] Pruett M.K., Cowan P.A., Cowan C.P., Gillette P., Pruett K.D. (2019). Supporting father involvement: an intervention with community and child welfare–referred couples. Fam Relat.

[74] Rincón P., Cova F., Saldivia S. (2018). Effectiveness of a positive parental practices training program for Chilean preschoolers' families: a randomized controlled trial. Front Psychol.

[75] Selby E., Allabyrne C., Keenan J.R. (2021). Delivering clinical evidence-based child–parent interventions for emotional development through a digital platform: a feasibility trial. Clin Child Psychol Psychiatr.

[76] Self-Brown S., Osborne M.C., Boyd C. (2018). The impact of SafeCare® Dads to Kids program on father maltreatment risk and involvement: outcomes and lessons learned from an efficacy trial. Child Abuse Negl.

[77] Silovsky J.F., Bard D., Chaffin M. (2011). Prevention of child maltreatment in high-risk rural families: a randomized clinical trial with child welfare outcomes. Child Youth Serv Rev.

[78] Sim A., Puffer E., Green E. (2014).

[79] Smith M.J. (2010).

[80] Solís Cámara P., Covarrubias Salcido P., Díaz Romero M., Rivera Aguirre B.I. (2004). Efectos multidimensionales de un programa de crianza en la interacción recíproca entre padres y sus niños pequeños con problemas de comportamiento. Psicol Conduct.

[81] Solís-Cámara P., Medina Cuevas Y., Díaz Romero M. (2015). Comparative analysis of predictive factors of severe disciplinary practices with preschoolers, before and after parent training. Acta Colomb de Psicol.

[82] Sourander A., McGrath P.J., Ristkari T. (2016). Internet-assisted parent training intervention for disruptive behavior in 4-year-old children: a randomized clinical trial. JAMA Psychiatr.

[83] Spaccarelli S., Cotler S., Penman D. (1992). Problem-solving skills training as a supplement to behavioral parent training. Cognit Ther Res.

[84] Villodas M.T., Moses J.O., Cromer K.D. (2021). Feasibility and promise of community providers implementing home-based parent-child interaction therapy for families investigated for child abuse: a pilot randomized controlled trial. Child Abuse Negl.

[85] Ward C.L., Wessels I.M., Lachman J.M. (2020). Parenting for lifelong health for young children: a randomized controlled trial of a parenting program in South Africa to prevent harsh parenting and child conduct problems. J Child Psychol Psychiatry.

[86] Webster-Stratton C. (1984). Randomized trial of two parent-training programs for families with conduct-disordered children. J Consult Clin Psychol.

[87] Webster-Stratton C. (1990). Enhancing the effectiveness of self-administered videotape parent training for families with conduct-problem children. J Abnorm Child Psychol.

[88] Wolfe D.A., Edwards B., Manion I., Koverola C. (1988). Early intervention for parents at risk of child abuse and neglect: a preliminary investigation. J Consult Clin Psychol.

[89] Yao A., Shimada K., Kasaba R., Tomoda A. (2022). Beneficial effects of behavioral parent training on inhibitory control in children with attention-deficit/hyperactivity disorder: a small-scale randomized controlled trial. Front Psychol.

[90] Zahra E.D., Nazanin V., Reza E.M., Sima K., Zohreh S. (2014). Implementation of mother-training program to improve parenting in pre-school age children: a randomized-controlled trial. N Am J Med Sci.

[91] Cohen L.J. (1977).

[92] Jeong J., Pitchik H.O., Fink G. (2021). Short-term, medium-term and long-term effects of early parenting interventions in low-and middle-income countries: a systematic review. BMJ Glob Health.

[93] Dishion T.J., Nelson S.E., Kavanagh K. (2003). The family check-up with high-risk young adolescents: preventing early-onset substance use by parent monitoring. Behav Ther.

[94] Perri M.G. (1998). The maintenance of treatment effects in the long-term management of obesity. Clin Psychol Sci Pract.

[95] Volpp K.G., Troxel A.B., Pauly M.V. (2009). A randomized, controlled trial of financial incentives for smoking cessation. N Engl J Med.

[96] Middleton K.R., Anton S.D., Perri M.G. (2013). Long-term adherence to health behavior change. Am J Lifestyle Med.

[97] Bouton M.E. (2014). Why behavior change is difficult to sustain. Prev Med.

[98] Wood W., Neal D.T. (2016). Healthy through habit: interventions for initiating & maintaining health behavior change. Behav Sci Pol.

[99] Michie S., Van Stralen M.M., West R. (2011). The behaviour change wheel: a new method for characterising and designing behaviour change interventions. Implement Sci.

[100] Stoltenborgh M., Bakermans-Kranenburg M.J., Alink L.R., van IJzendoorn M.H. (2015). The prevalence of child maltreatment across the globe: review of a series of meta-analyses. Child Abuse Rev.

[101] Lipsey M.W. (2003). Those confounded moderators in meta-analysis: good, bad, and ugly. Ann Am Acad Pol Soc Sci.

