# Supplementary material for: Effects over time of parenting interventions to reduce physical and emotional violence against children: a systematic review and meta-analysis
Source: eClinicalMedicine. 2023 May 18;60:102003. doi: 10.1016/j.eclinm.2023.102003 (PMC10209692; doi:10.1016/j.eclinm.2023.102003)
Supplement: Supplementary Figs. S1–S5 and Tables S1 and S2 [file mmc1.docx]

**Online supplement**

**Table S1. Eligibility criteria for inclusion**

| ***Domain*** | **Inclusion criteria** | **Exclusion criteria** |
| --- | --- | --- |
| *Population* | Parents and other caregivers of children with a mean age between 2 and 10 years.  If studies did not report the mean value, two steps were taken: a. contacting the author(s) to ask for the mean age; b. if the observed mean value remained unclear, a theoretical mean was calculated by using the minimum and maximum age value and dividing it by two. | We exclude trials specifically aimed at special groups such as physical, learning or developmental disabilities, children with severe mental illness (ADHD is included, as long as the study explicitly focused on reducing conduct problems), and children in temporary foster care. We exclude adults providing care to children in institutional settings. |
| *Intervention* | Parenting intervention with at least 50% of sessions or content directed at parents. The intervention has a clear theoretical foundation largely comprising social learning theory principles.  Social learning theory posits that children develop disruptive behaviour when parents unwittingly reward disruptive behaviour instead of positive behaviour, and when parents model aversive behaviour. Interventions based on social learning theory teach parents behaviour management skills such as setting clear rules, using positive reinforcement and preventing negative reinforcement, and finding alternatives to harsh and abusive parenting.  We used the following steps to ensure that an intervention is based on social learning theoretical principles:  1. We read the intervention description of the trial paper and searched for terms such as social learning or coercive parent-child interactions.  2. We searched for additional publications by the intervention developer that describe the intervention.  3. We retrieved the manuals of the interventions and checked the components. At least 50% of the components had to focus on redirecting child behaviours through differential attention or parental modelling of non-violent behaviours (for example: using time-out, praise, rewards, rule setting, ignoring, modelling);  4. We searched for policy briefs or other online resources that describe the intervention | We exclude interventions that focus mainly (more than 50% of sessions or content) on specific aspects of parenting, such as toileting, sexual health, feeding or HIV prevention, rather than teaching general parenting skills.  In addition, we exclude interventions which:  a. focus narrowly on very specific child risks such as poisoning or accidents, or which teach skills for dealing with specific medical conditions or physical disabilities, such as asthma, epilepsy, HIV, psychosis, autism, Down Syndrome or severe learning disabilities;  b. primarily deliver financial, social or other support to parents but do not aim to change parents’ knowledge or behaviour (e.g. conditional cash transfer programs, unless they include a parent training component, the effects of which can be analysed separately from other components). |
| *Comparator* | Inactive control groups (no treatment, waiting list, minimal intervention, treatment as usual) | We exclude studies/study arms with an active condition such as a variant of the same parenting intervention, a different parenting intervention or an alternative intervention. |
| *Outcomes* | This systematic review aimed to answer effectiveness of parenting interventions on a range of outcomes. For this study, we include measures that use systematic direct observational techniques, instruments that rely on self-report and, if available, official reports of maltreatment.  At least 50% of items of a subscale or instrument needed to include physically or emotionally violent parenting behaviours. For observational measures, coding example needed to clearly indicate emotional or physical violence. This outcome was renamed to physical and emotional violence (see online protocol). | We excluded outcomes that included any other form of violence including neglect, and instruments that had less than 50% of violent items. |
| *Study design* | Randomised controlled trials and cluster-randomised controlled trials |  |

**Table S2. Detailed GRADE rating**

**Author(s):** Backhaus S, Leijten P, Jochim J, Melendez-Torres, G.J., Gardner F.

**Question:** How effective are parenting interventions based on social learning theory for parents and caregivers of children aged 2 to 10 years compared to an inactive control condition for reducing emotional and physical violence?

**Setting:** Global

| **Certainty assessment** | | | | | | | **№ of patients** | | **Effect** | | **Certainty** | **Importance** |
| --- | --- | --- | --- | --- | --- | --- | --- | --- | --- | --- | --- | --- |
| **№ of studies** | **Study design** | **Risk of bias** | **Inconsistency** | **Indirectness** | **Imprecision** | **Other considerations** | **Parenting intervention** | **Inactive control group** | **Relative (95% CI)** | **Absolute (95% CI)** |  |  |
| Immediate effects | | | | | | | | | | | | |
| 42 | randomised trials | serious^a^ | not serious | not serious | not serious | none | 2388 | 2062 | - | SMD **0·46 lower** (0·59 lower to 0·33lower) | ⨁⨁⨁◯ Moderate | CRITICAL |
| 1-6 months effects | | | | | | | | | | | | |
| 18 | randomised trials | very serious^b^ | not serious | not serious | not serious | none | 1901 | 1703 | - | SMD **0·24 lower** (0·37 lower to 0·11 lower) | ⨁⨁◯◯ Low | CRITICAL |
| 7-24 months effects | | | | | | | | | | | | |
| 12 | randomised trials | serious^c^ | not serious | not serious | not serious | publication bias strongly suspected^d^ | 1264 | 1135 | - | SMD **0·18 lower** (0·34 lower to 0·02 lower) | ⨁⨁◯◯ Low | CRITICAL |

**CI:** confidence interval; **SMD:** standardized mean difference

#### Explanations

a. Majority of studies in the body of evidence at a high risk of bias largely due to unclear risks for key domains.

b. Majority of studies in the body of evidence at a high risk of bias largely due to high risk of blinding of outcome assessors which was not a key domain, and high risk of bias for selected outcome reporting

c. Majority of studies in the body of evidence at a high risk of bias largely due to high risk of blinding of outcome assessors which was not a key domain.

d. Small trials in the body of evidence report no effects in the opposite direction.

**Figure S1. Funnel plot of intervention studies with effects on physical and emotional violence at immediate post-test**


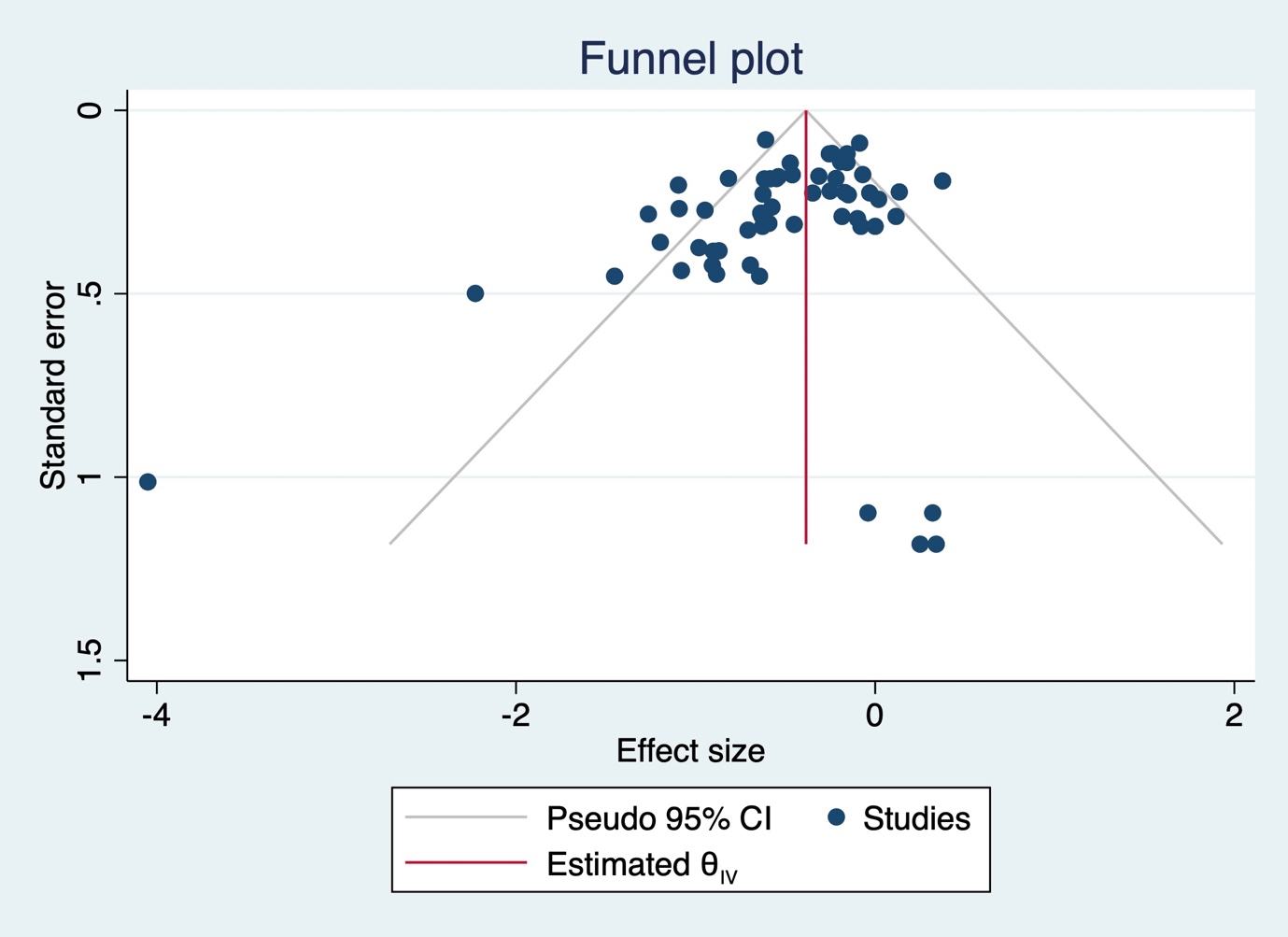


**Figure S2. Funnel plot of intervention studies with effects on physical and emotional violence at short-term follow-up**


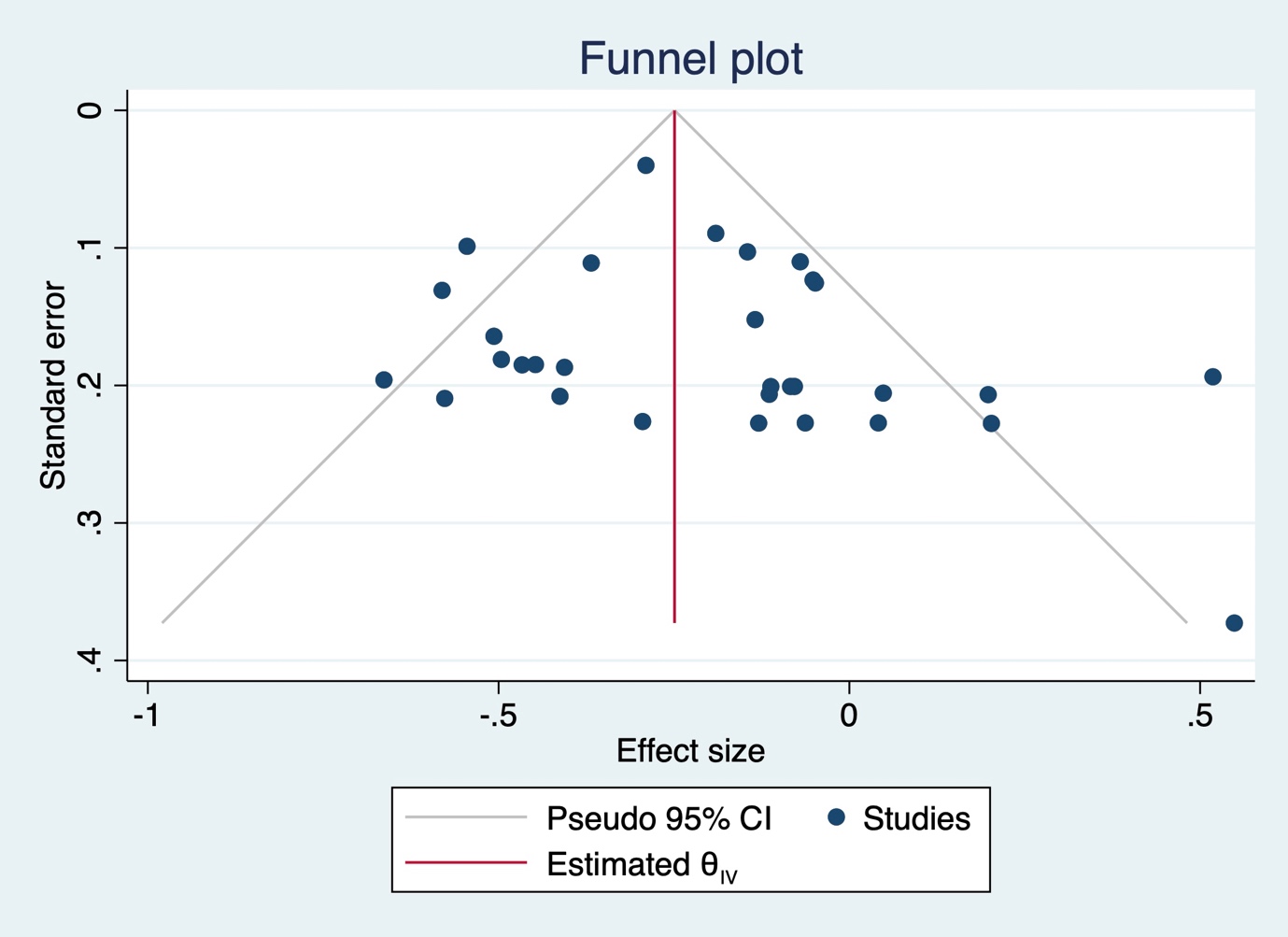


**Figure S3. Funnel plot of intervention studies with effects on physical and emotional violence at longer-term follow-up**


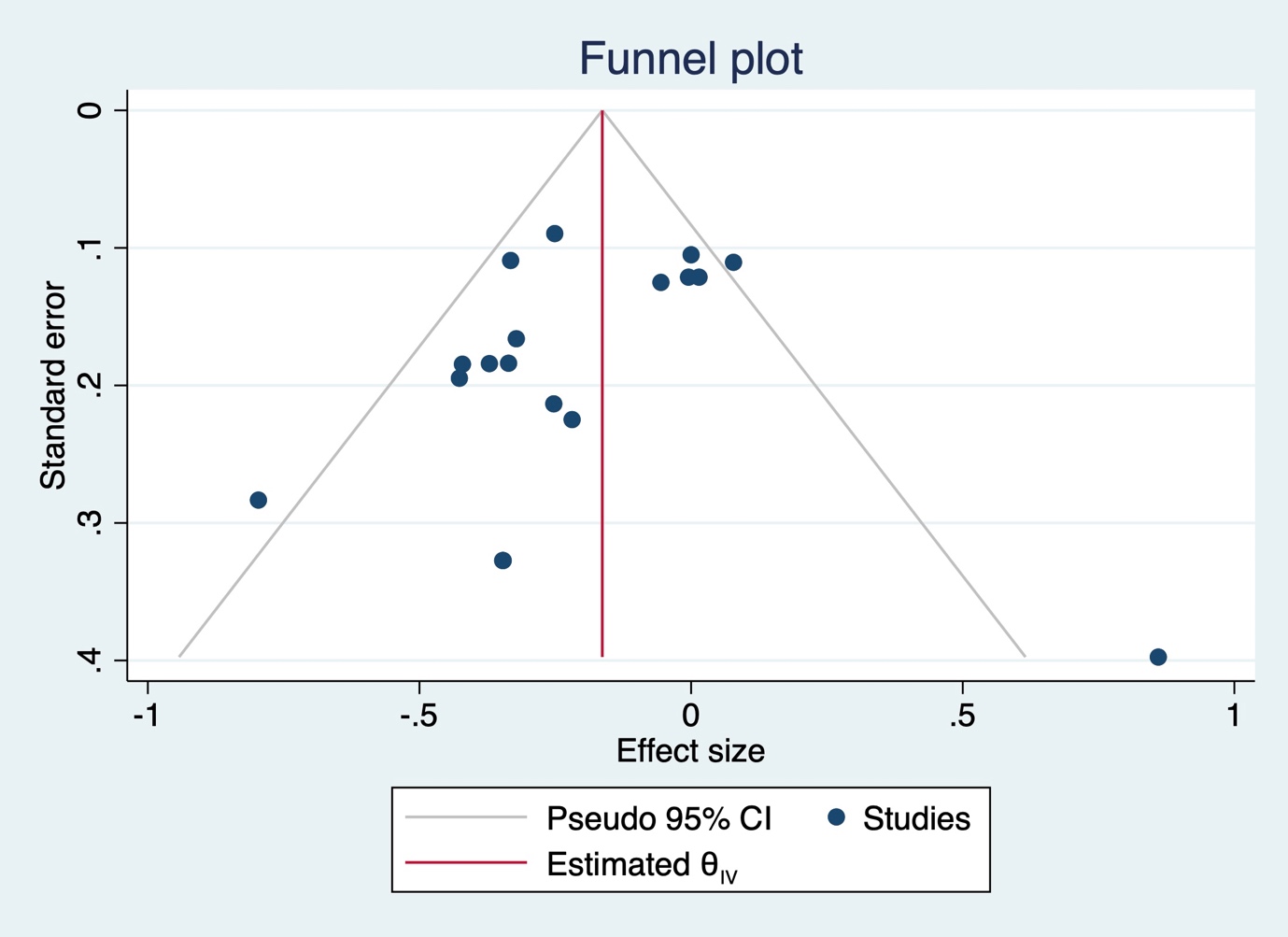


**Figure S4. Detailed risk of bias assessment**


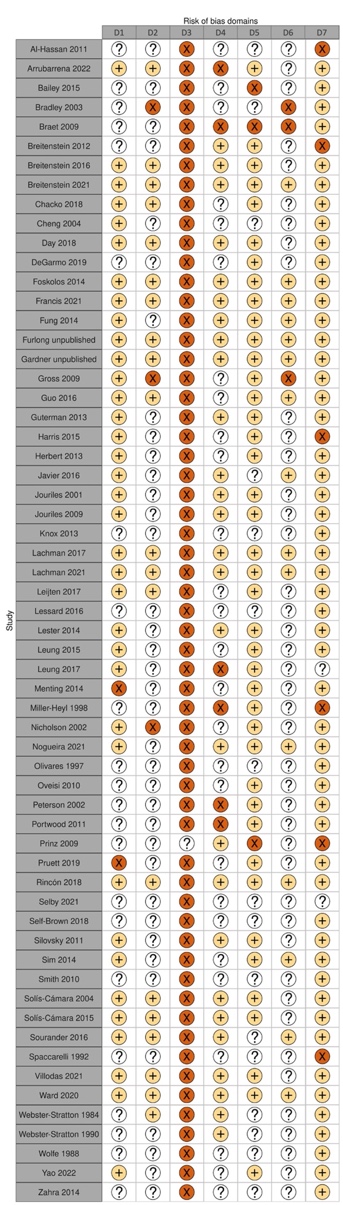


D1= Sequence allocation, D2 = Allocation concealment, D3 = Blinding of participants, D4= Blinding of outcome assessors, D5 = Incomplete data assessment, D6 = Selected outcome reporting, D7 = Other bias

- = Low risk ;
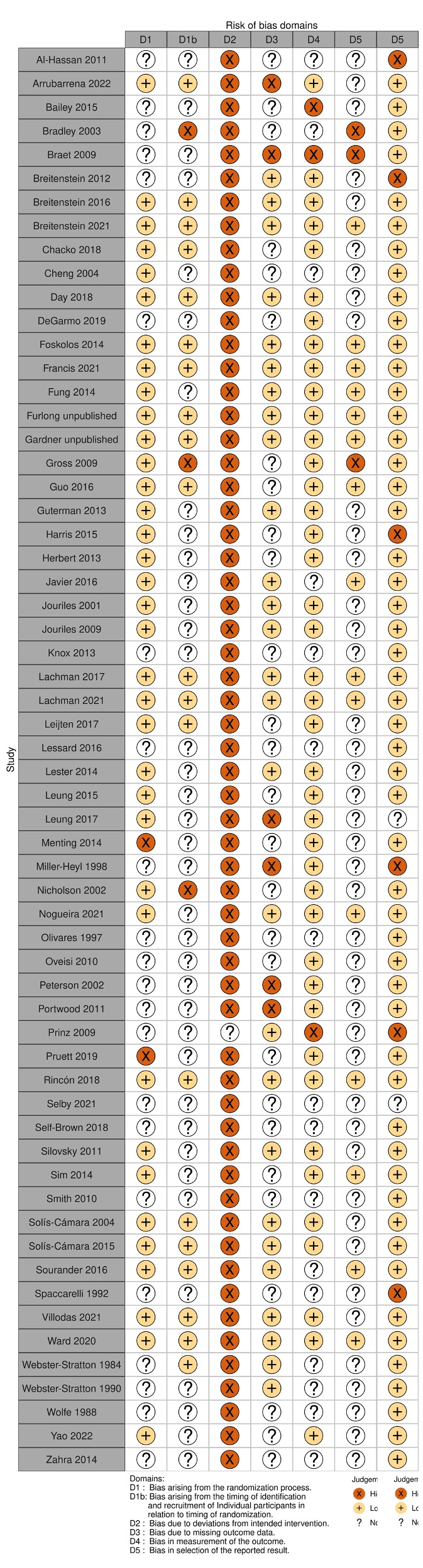
 = Unclear risk;
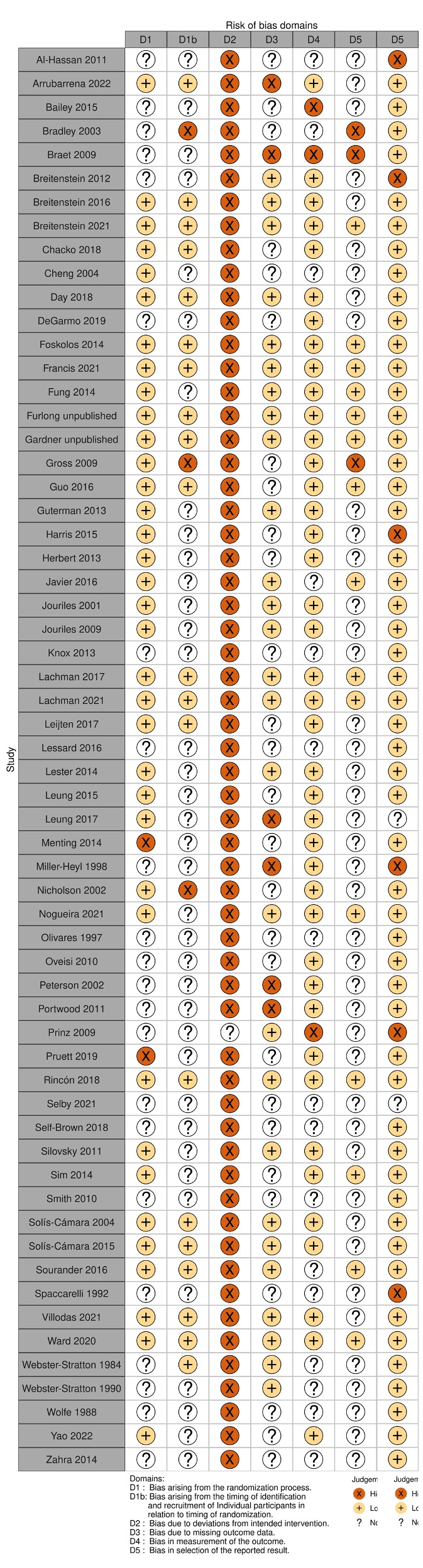
 = High risk

**Figure S5. Histogram of number of effect sizes by measurement time point**

**
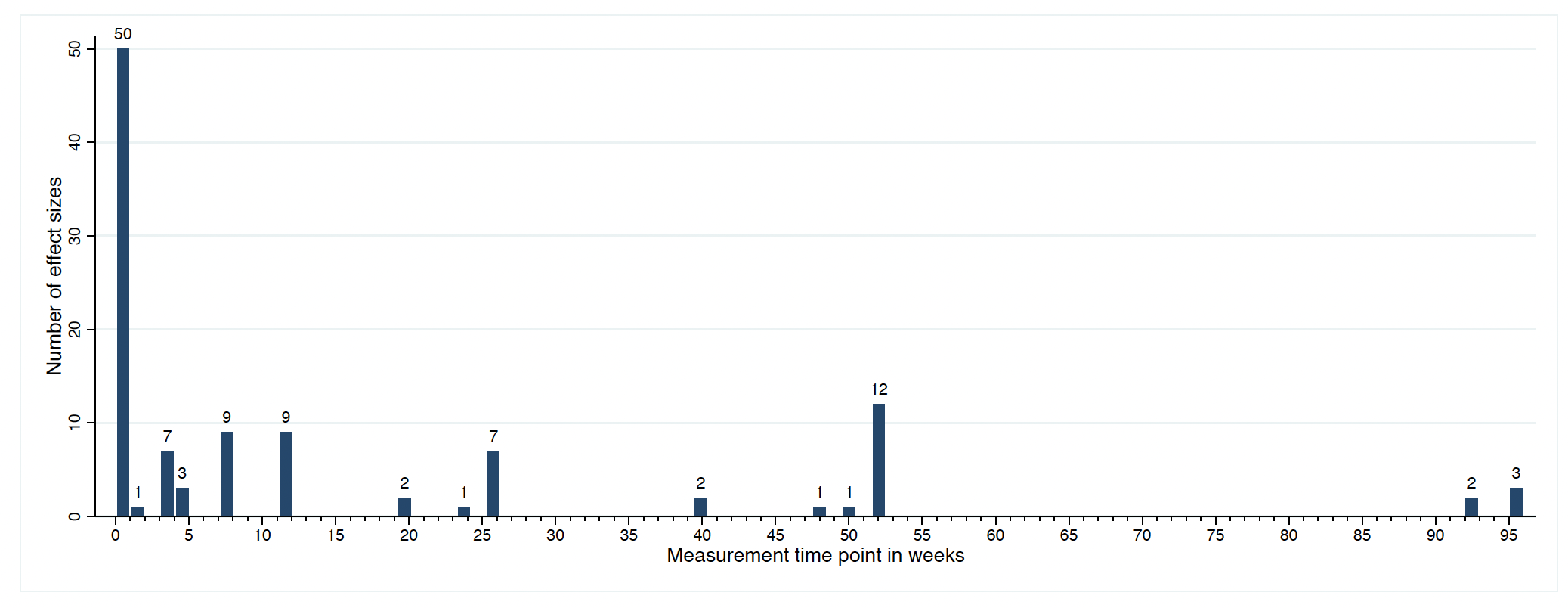
**

**Search terms:**

We took the following essential concepts of the inclusion criteria to develop the search string. These terms were combined using Boolean logic (“or”, “and”). Four conceptual categories with relevant terms were used: 1) intervention, 2) parenting (including abuse), 3) child behavioural and emotional problems, 4) we did not use a term for RCT, as there is evidence that too many RCTs are missed by employing terms for the study design (Higgins & Green, 2008)

As an example, the search terms for MEDLINE:

1. ((parent$ or famil$) adj (program$ or intervention$ or training or education or group)).tw.

2. behavior therapy/ or cognitive therapy/

3. (behavio#r adj3 (train$ or intervention$ or therap$ or program$)).tw.

4. (cbt or cognitive behavio#ral therapy).tw.

5. (cognitive adj3 (therap$ or intervention$ or train$ or program$)).tw.

6. (triple p or positive parenting program).ti,ab,kw.

7. incredible years.ti,ab,kw.

8. PCIT.mp. or (Parent-child adj interaction adj therap$).ti,ab,kw. [mp=title, abstract, original title, name of substance word, subject heading word, floating sub-heading word, keyword heading word, organism supplementary concept word, protocol supplementary concept word, rare disease supplementary concept word, unique identifier, synonyms]

9. PMT.mp. or (parent adj management adj training).ti,ab,kw. [mp=title, abstract, original title, name of substance word, subject heading word, floating sub-heading word, keyword heading word, organism supplementary concept word, protocol supplementary concept word, rare disease supplementary concept word, unique identifier, synonyms]

10. (family adj check-up).ti,ab,kw.

11. 1 or 2 or 3 or 4 or 5 or 6 or 7 or 8 or 9 or 10

12. conduct disorder$.mp.

13. (oppositional adj3 (defiant$ or disorder$)).mp.

14. (conduct adj3 (difficult$ or disorder$ or problem$)).mp.

15. (behavio#ral adj3 (problem$ or difficult$ or disorder$)).mp.

16. aggressive behavio#r$.mp.

17. (emotional adj1 behavio#ral problem$).mp.

18. (child$ adj3 behavio#r$ disorder$).mp.

19. social behavio#r disorder$.mp.

20. ((antisocial or externali$ or internali$ or disruptive) adj (behavio#r or problem$ or difficult$)).mp. [mp=title, abstract, original title, name of substance word, subject heading word, floating sub-heading word, keyword heading word, organism supplementary concept word, protocol supplementary concept word, rare disease supplementary concept word, unique identifier, synonyms]

21. ((child adj abus$) or maltreat$ or (psychological adj aggression) or neglect or (corporal adj punish$)).mp.

22. ((exp parenting skills/ or exp disciplin$/ or exp emotio$/) adj regulation/) or exp warmth/ or parenting/ or exp Mother Child Communica$/ or exp Child Disciplin$/ or exp Father Child Relation$/ or exp Mother Child Relation$/ or exp Parent Child Relation$/ or exp Parent Child Communicati$/ or exp Father Child Communicat$/ or exp child parent relation$/ or exp child rearing/ or exp family functioning/ or exp family conflict/ or exp maternal behavio#r/ or exp paternal behavio#r/

23. 12 or 13 or 14 or 15 or 16 or 17 or 18 or 19 or 20 or 21 or 22

24. 11 and 23

25. limit 24 to yr="2014 -Current"


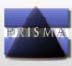
**PRISMA 2020 Checklist**

| **Section and Topic** | **Item #** | **Checklist item** | **Location where item is reported** |
| --- | --- | --- | --- |
| **TITLE** | | |  |
| Title | 1 | Identify the report as a systematic review. | Page 1 |
| **ABSTRACT** | | |  |
| Abstract | 2 | See the PRISMA 2020 for Abstracts checklist. | Page 1 |
| **INTRODUCTION** | | |  |
| Rationale | 3 | Describe the rationale for the review in the context of existing knowledge. | Pages 1–2 |
| Objectives | 4 | Provide an explicit statement of the objective(s) or question(s) the review addresses. | Page 3 |
| **METHODS** | | |  |
| Eligibility criteria | 5 | Specify the inclusion and exclusion criteria for the review and how studies were grouped for the syntheses. | Page 3 & supplement |
| Information sources | 6 | Specify all databases, registers, websites, organisations, reference lists and other sources searched or consulted to identify studies. Specify the date when each source was last searched or consulted. | Page 3 |
| Search strategy | 7 | Present the full search strategies for all databases, registers and websites, including any filters and limits used. | Supplement |
| Selection process | 8 | Specify the methods used to decide whether a study met the inclusion criteria of the review, including how many reviewers screened each record and each report retrieved, whether they worked independently, and if applicable, details of automation tools used in the process. | Page 3 |
| Data collection process | 9 | Specify the methods used to collect data from reports, including how many reviewers collected data from each report, whether they worked independently, any processes for obtaining or confirming data from study investigators, and if applicable, details of automation tools used in the process. | Page 3 |
| Data items | 10a | List and define all outcomes for which data were sought. Specify whether all results that were compatible with each outcome domain in each study were sought (e.g. for all measures, time points, analyses), and if not, the methods used to decide which results to collect. | Page 3 and supplement |
|  | 10b | List and define all other variables for which data were sought (e.g. participant and intervention characteristics, funding sources). Describe any assumptions made about any missing or unclear information. | Page 3 |
| Study risk of bias assessment | 11 | Specify the methods used to assess risk of bias in the included studies, including details of the tool(s) used, how many reviewers assessed each study and whether they worked independently, and if applicable, details of automation tools used in the process. | Page 3 |
| Effect measures | 12 | Specify for each outcome the effect measure(s) (e.g. risk ratio, mean difference) used in the synthesis or presentation of results. | Pages 3–4 |
| Synthesis methods | 13a | Describe the processes used to decide which studies were eligible for each synthesis (e.g. tabulating the study intervention characteristics and comparing against the planned groups for each synthesis (item #5)). | Page 4 |
|  | 13b | Describe any methods required to prepare the data for presentation or synthesis, such as handling of missing summary statistics, or data conversions. | Page 4 |
|  | 13c | Describe any methods used to tabulate or visually display results of individual studies and syntheses. | Page 4 |
|  | 13d | Describe any methods used to synthesize results and provide a rationale for the choice(s). If meta-analysis was performed, describe the model(s), method(s) to identify the presence and extent of statistical heterogeneity, and software package(s) used. | Pages 4 |
|  | 13e | Describe any methods used to explore possible causes of heterogeneity among study results (e.g. subgroup analysis, meta-regression). | Page 4 |
|  | 13f | Describe any sensitivity analyses conducted to assess robustness of the synthesized results. | Page 5 |
| Reporting bias assessment | 14 | Describe any methods used to assess risk of bias due to missing results in a synthesis (arising from reporting biases). | Page 4 |
| Certainty assessment | 15 | Describe any methods used to assess certainty (or confidence) in the body of evidence for an outcome. | Page 4 |
| **RESULTS** | | |  |
| Study selection | 16a | Describe the results of the search and selection process, from the number of records identified in the search to the number of studies included in the review, ideally using a flow diagram. | Figure 1 |
|  | 16b | Cite studies that might appear to meet the inclusion criteria, but which were excluded, and explain why they were excluded. | - |
| Study characteristics | 17 | Cite each included study and present its characteristics. | Tables 1&2 |
| Risk of bias in studies | 18 | Present assessments of risk of bias for each included study. | Supplement Figure S4 |
| Results of individual studies | 19 | For all outcomes, present, for each study: (a) summary statistics for each group (where appropriate) and (b) an effect estimate and its precision (e.g. confidence/credible interval), ideally using structured tables or plots. | Figures 3-5 |
| Results of syntheses | 20a | For each synthesis, briefly summarise the characteristics and risk of bias among contributing studies. | Table 3 & Table S2 |
|  | 20b | Present results of all statistical syntheses conducted. If meta-analysis was done, present for each the summary estimate and its precision (e.g. confidence/credible interval) and measures of statistical heterogeneity. If comparing groups, describe the direction of the effect. | Table 3 |
|  | 20c | Present results of all investigations of possible causes of heterogeneity among study results. | Page 5 |
|  | 20d | Present results of all sensitivity analyses conducted to assess the robustness of the synthesized results. | Page 5 |
| Reporting biases | 21 | Present assessments of risk of bias due to missing results (arising from reporting biases) for each synthesis assessed. | Funnel plots Figures S1-3 |
| Certainty of evidence | 22 | Present assessments of certainty (or confidence) in the body of evidence for each outcome assessed. | Table 3 & Table S2. |
| **DISCUSSION** | | |  |
| Discussion | 23a | Provide a general interpretation of the results in the context of other evidence. | Page 9 |
|  | 23b | Discuss any limitations of the evidence included in the review. | Pages 11–12 |
|  | 23c | Discuss any limitations of the review processes used. | Pages 11–12 |
|  | 23d | Discuss implications of the results for practice, policy, and future research. | Pages 2, 12 |
| **OTHER INFORMATION** | | |  |
| Registration and protocol | 24a | Provide registration information for the review, including register name and registration number, or state that the review was not registered. | Pages 1, 4 |
|  | 24b | Indicate where the review protocol can be accessed, or state that a protocol was not prepared. | Pages 1, 4 |
|  | 24c | Describe and explain any amendments to information provided at registration or in the protocol. | Table S1 |
| Support | 25 | Describe sources of financial or non-financial support for the review, and the role of the funders or sponsors in the review. | Pages 1, 4 13 |
| Competing interests | 26 | Declare any competing interests of review authors. | Page 13 |
| Availability of data, code and other materials | 27 | Report which of the following are publicly available and where they can be found: template data collection forms; data extracted from included studies; data used for all analyses; analytic code; any other materials used in the review. | Page 13 |

*From:*  Page MJ, McKenzie JE, Bossuyt PM, Boutron I, Hoffmann TC, Mulrow CD, et al. The PRISMA 2020 statement: an updated guideline for reporting systematic reviews. BMJ 2021;372:n71. doi: 10.1136/bmj.n71
